# Long-chain fatty acyl coenzyme A inhibits NME1/2 and regulates cancer metastasis

**DOI:** 10.1073/pnas.2117013119

**Published:** 2022-03-08

**Authors:** Shuai Zhang, Ornella D. Nelson, Ian R. Price, Chengliang Zhu, Xuan Lu, Irma R. Fernandez, Robert S. Weiss, Hening Lin

**Affiliations:** ^a^Howard Hughes Medical Institute, Cornell University, Ithaca, NY 14853;; ^b^Department of Chemistry and Chemical Biology, Cornell University, Ithaca, NY 14853;; ^c^Department of Biomedical Sciences, Cornell University, Ithaca, NY 14853

**Keywords:** long-chain fatty acyl coenzyme A, fatty acid, tumor metastasis, NME1, NME2

## Abstract

Fatty acid metabolism has well-established connections to cancer progression and metastasis. However, whether the metabolic intermediates of fatty acid metabolism regulate this process through protein–metabolite interactions remains largely unknown. Here, we investigated whether long-chain fatty acyl coenzyme A (LCFA-CoA), an important metabolic intermediate involved in fatty acid metabolism, could regulate cellular protein functions to affect cancer. We used a quantitative chemical proteomic approach to identify proteins that could be regulated by LCFA-CoA. This strategy identified NME1 and NME2 as potential targets regulated by LCFA-CoA. In vitro, LCFA-CoA potently inhibited NME1/2. In cells, LCFA-CoA inhibited clathrin-mediated endocytosis and cancer cell migration, processes regulated by NME1/2. In vivo, NME1, a known metastasis suppressor, is inhibited by LCFA-CoA, and its metastasis suppressor function is compromised in mouse models of breast cancer specifically under high-fat–diet conditions. Thus, inhibition of NME1 by LCFA-CoA provides a molecular mechanism linking fatty acid metabolism and cancer metastasis, demonstrating the power of the chemical proteomic approach for discovering regulatory roles of metabolites.

## Significance

The study provided a long-sought molecular mechanism that could explain the link between fatty acid metabolism and cancer metastasis. Further understanding may lead to new strategies to inhibit cancer metastasis. The chemical proteomic approach developed here will be useful for discovering other regulatory mechanisms of protein function by small molecule metabolites.

Beyond serving as intermediates in various metabolic pathways, metabolites can also regulate various biological pathways to help maintain proper cellular and organismal functions (1, 2). Such regulatory functions can be achieved through protein posttranslational modifications (PTMs), as many PTMs, such as phosphorylation, acetylation, and methylation, use metabolites as cosubstrates. Functional regulation can also be achieved through protein–metabolite noncovalent interactions. Compared with the rapid progress in PTM research and the high appreciation of the regulatory roles of PTMs, understanding of the noncovalent regulatory roles of metabolites is lagging far behind. Here, we use a quantitative chemical proteomic approach to systematically identify proteins that can be bound and regulated by long-chain fatty acyl coenzyme A (LCFA-CoA). LCFA-CoAs are important metabolites in lipid biosynthesis and fatty acid degradation (3). Although LCFA-CoAs are reported to regulate multiple enzymes in vitro, in vivo evidence for important regulatory functions of LCFA-CoAs is lacking (4). A recent study found that LCFA-CoA can be sensed by AMP-activated protein kinase and may serve as an indicator for lipid energy source availability, suggesting broader regulatory functions of LCFA-CoA (5).

Increased fatty acid synthesis is one of the most obvious metabolic changes in tumor cells. Reprogramming of fatty acid metabolism in cancer can promote tumor growth, angiogenesis, survival, and metastasis (6). However, it is not clear whether the change in fatty acid metabolism is simply to meet the increased metabolic demands for fatty acids or if the increase in intermediates from fatty acids metabolism could contribute to tumor progression by regulating protein functions. Because most metabolic pathways for fatty acids require the formation of LCFA-CoA, we suspected that LCFA-CoA may play important roles in regulating protein function and thus promoting cancer progression. To test this hypothesis, we used a quantitative chemical proteomic strategy to profile proteins that bind to LCFA-CoA. We found that NME1, which is a metastasis suppressor (7, 8), can be inhibited by LCFA-CoA, highlighting a molecular mechanism that may impact how fatty acid metabolism affects breast cancer metastasis.

## Results

### Quantitative Chemical Proteomics Reveals Proteins That Are Potentially Regulated by LCFA-CoA.

**Fig. 1.**
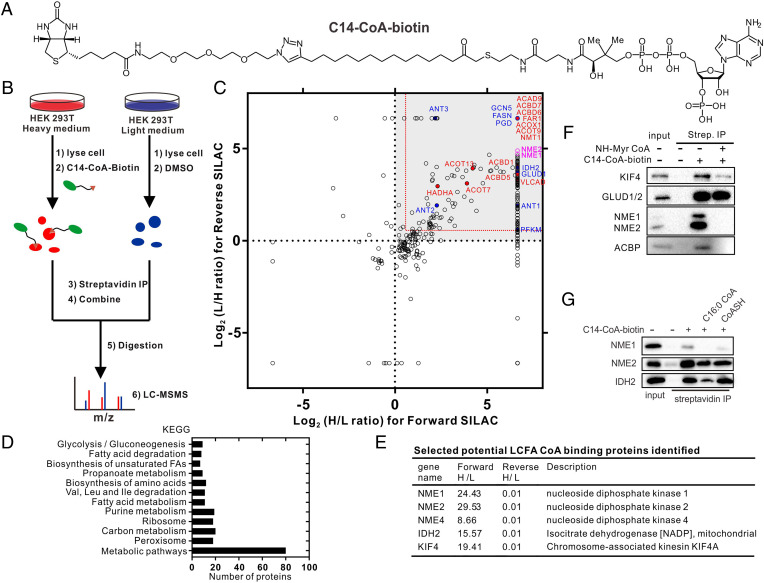
Quantitative chemical proteomics reveals potential LCFA-CoA–binding proteins. (*A*) Structure of C14-CoA-biotin probe. (*B*) Workflow for SILAC experiments to profile LCFA-CoA–binding proteins. (*C*) Results of the SILAC experiments with HEK293T cell lysates. Scatterplot of log_2_(H/L ratio) of identified proteins in forward SILAC against log_2_(L/H ratio) of proteins identified in reverse SILAC. Proteins with >1.5-fold enrichment (red dashed line) in both forward and reverse SILAC were considered as possible LCFA-CoA interacting proteins. Red spots are proteins known to use LCFA-CoA as substrates, blue spots are proteins reported to be regulated by LCFA-CoA in vitro, and NME1/2 are in pink. (*D*) Biological process analysis of the LCFA-CoA–binding proteins. (*E*) Partial list of the top hits from the SILAC experiment. (*F*) Validation of some identified proteins by C14-CoA-biotin pull-down and Western blot in cell lysate. Nonhydrolyzable myristoyl CoA (NH-Myr-CoA) is used as a competitor. (*G*) Validation of some identified proteins by C14-CoA-biotin probe pull-down and Western blot with purified proteins. C16:0 CoA or CoASH was used as a competitor. LC-MSMS, liquid chromatography-tandem mass spectrometry; Strep., streptavidin; IP, immunoprecipation; FA, fatty acid; CoASH, free coenzyme A.

To profile the binding partners of LCFA-CoA, we designed and synthesized a C14-CoA-biotin probe (Fig. 1*A*) that enabled the pull-down of LCFA-CoA–binding proteins from cell lysate. To avoid the breakdown of the C14-CoA-biotin probe during the experiment and increase pull-down efficiency, we used a nonhydrolyzable, methylene-bridged analog of myristoyl-CoA. The biotin tag enables the affinity pull-down of proteins bound to the LCFA-CoA analog. To increase the reliability of the proteomic result, we applied the probe in SILAC (stable isotope labeling by amino acids in cell culture) experiments to profile LCFA-CoA–binding proteins (Fig. 1*B*). In a forward SILAC experiment, C14-CoA-biotin probe was added in the heavy-labeled cell lysate as a bait for LCFA-CoA–binding proteins, whereas DMSO (dimethyl sulfoxide) was added in an equal amount of light cell lysate as a control. In a reverse SILAC experiment, C14-CoA-biotin probe was added in the light cell lysate and DMSO was added in the heavy-labeled cell lysate. After 1-h incubation at 4 °C, streptavidin pull-down was conducted in both heavy and light samples. The heavy and light pull-down fractions were mixed, trypsin digested, and analyzed with mass spectrometry. Only proteins with high heavy/light (H/L) ratios >1.5 in the forward experiment and <0.67 in the reverse experiment were considered as possible LCFA-CoA–interacting proteins. A list of all identified proteins is given in Dataset S1.

To visualize the results, we generated a scatterplot of log_2_(forward H/L ratio) against log_2_(reverse L/H ratio) of all identified proteins (Fig. 1*C*). Proteins that may bind to LCFA-CoA were clustered in the upper right quadrant. In total, we identified 317 proteins that potentially bind to LCFA-CoA, of which 13 are known to use LCFA-CoA as substrate (red circles in Fig. 1*C* and *SI Appendix*, Table S1) and 8 are reported to be regulated by LCFA-CoA in vitro (blue circles in Fig. 1*C* and *SI Appendix*, Table S2). The identification of these proteins suggests that the proteomic result is reliable.

For our downstream analysis, we focused on identified proteins that have not been known to use LCFA-CoA as a substrate or to be regulated by LCFA-CoA. Most of these proteins are involved in metabolic processes, indicating a potential regulatory function of LCFA-CoA in metabolism (Fig. 1*D* and *SI Appendix*, Fig. S1). Three proteins caught our attention, NME1, NME2, and NME4 (Fig. 1*E*), which are members of the nucleoside diphosphate kinase (NDPK) family. The major function of NDPKs is catalyzing the transfer of a phosphate group from nucleoside triphosphates, mainly adenosine triphosphate (ATP), to nucleoside diphosphates, in particular guanosine diphosphate (GDP), through a ping-pong mechanism involving the formation of a phosphohistidine intermediate (9, 10). NME1, NME2, and NME4, which share high levels of homology, form hexamers as the catalytically active form (8, 11). NME proteins have been shown to have other enzymatic activities and be involved in different physiological and pathological processes (12). NME1 was the first identified metastasis suppressor gene (7), and an inverse association between NME1 expression and metastatic ability has been observed in several solid tumor types including melanoma and breast, colon, lung, liver, ovary, prostate, and oral carcinomas (13–19).

To validate that NME indeed can bind to LCFA-CoA, we used nonhydrolyzable myristoyl CoA as a competitor in the pull-down experiment in HEK293T cell lysate (Fig. 1*F* and *SI Appendix*, Fig. S2). As expected, NME1 and NME2 were pulled down by the C14-CoA-biotin probe, and this could be competed out by the nonhydrolyzable myristoyl CoA. Purified preparations of additional proteins identified in the SILAC proteomics were also confirmed to bind to LCFA-CoA in this assay (Fig. 1*G*). The binding of NME1/2 to 25 μM of the probe was competed more effectively by 100 μM of palmitoyl CoA than by 1 mM of free CoA, indicating that NME proteins bind LCFA-CoA directly and tightly (Fig. 1*G*).

### LCFA-CoA Inhibits NDPKs In Vitro.

**Fig. 2.**
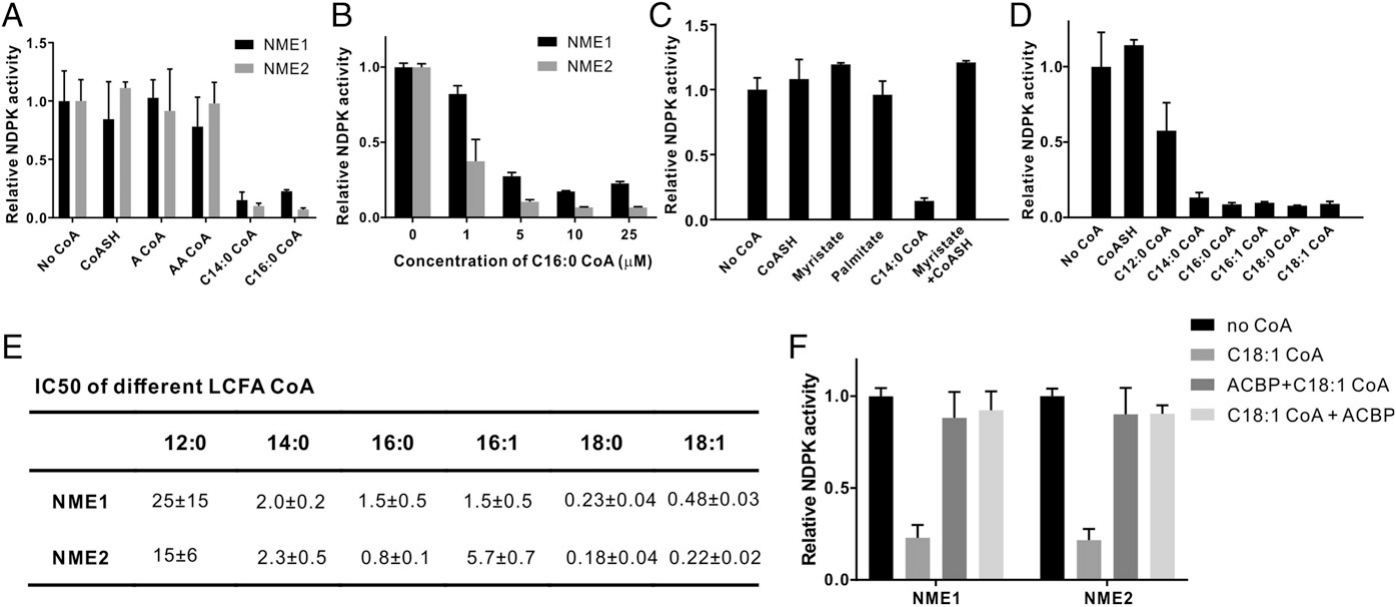
LCFA-CoA esters inhibit NDPKs in vitro. (*A*) LCFA-CoA but not other CoA molecules inhibit NME1 and NME2 in vitro. (*B*) Palmitoyl CoA inhibits NME1 and NME2 dose dependently in vitro. (*C*) LCFA-CoA, but not fatty acid or CoA, inhibits NME2 in vitro. (*D*) NME2 is inhibited by LCFA-CoA with different chain length. (*E*) IC_50_ of LCFA-CoA on NME proteins. (*F*) ACBP prevented and reversed the inhibition of NME1/2 by LCFA-CoA. C18:1 LCFA-CoA and ACBP concentrations were both 5 μM. For ACBP+C18:1 CoA experiment, NME was treated with ACBP first, then C18:1 CoA. In the C18:1 CoA+ACBP experiment, NME was treated with C18:1 CoA first, then ACBP. For all graphs, the bar heights reflect the mean and the error bars represent SD of independent replicates. A CoA, acetyl Coenzyme A; AA CoA, acetoacetyl coenzyme A; CoASH, free coenzyme A.

We next sought to examine the consequence of LCFA-CoA binding on the enzymatic activity of NME proteins. As NDPKs, the well-known function of NME1 and NME2 is to make guanosine triphosphate (GTP) from GDP and ATP to maintain intracellular nucleotide homeostasis. We optimized a high performance liquid chromatography–based in vitro enzymatic assay to monitor activity directly (*SI Appendix*, Fig. S3) and examined whether different CoA molecules could affect the NDPK activity of NME1 or NME2 in vitro. Among all the CoA derivatives tested, myristoyl CoA and palmitoyl CoA potently inhibited the NDPK activity of NME1 and NME2 while free CoA and short-chain acyl CoA molecules had no significant effect (Fig. 2*A*). The inhibition was dose dependent (Fig. 2*B*). Free CoA and fatty acid added together did not inhibit NME activity (Fig. 2*C*), indicating that LCFA-CoA is necessary for the inhibition.

We next examined the chain-length dependency of the inhibition and found that all saturated or monounsaturated long-chain (>14 carbons) fatty acyl CoA tested potently inhibited NDPK activity, while lauroyl CoA (C12:0 CoA, a medium-chain fatty acyl CoA) only inhibited moderately (Fig. 2*D*). We measured the half-maximal inhibitory concentration (IC_50_) values of different LCFA-CoA on both NME1 and NME2 (Fig. 2*E* and *SI Appendix*, Figs. S4 and S5). All the IC_50_ values were in the low micromolars or high nanomolars, which is within the physiological concentration range of LCFA-CoA (4, 20).

We also examined NME inhibition in the presence of acyl-CoA binding protein (ACBP), which has a high affinity for LCFA-CoAs (*K*_d_ ∼1 to 10 nM) (4). We found that an equal amount of ACBP almost completely protected NME1/2 from inhibition by C18:1 LCFA-CoA (Fig. 2*F*). ACBP could also reverse the NDPK activity of C18:1 LCFA-CoA–treated NME1/2 (Fig. 2*F*), which indicates the reversibility of the inhibition. Interestingly, the inhibition of NME by LCFA-CoA was dependent on the molar ratio of LCFA-CoA/ACBP, which is close to 1 in cell (21). When the LCFA-CoA/ACBP ratio was lower than 1, LCFA-CoA was sequestered and NME remained active. When the LCFA-CoA/ACBP ratio was higher than 1, NME was potently inhibited by LCFA-CoA (*SI Appendix*, Fig. S6).

Together, these results show that LCFA-CoA can potently inhibit NME1 and NME2 in vitro. The IC_50_ values of LCFA-CoA were within physiological ranges, indicating that this inhibition may happen in vivo, especially when LCFA-CoA/ACBP ratios are perturbed, such as under a high-fat diet (HFD) or obesity.

### Crystal Structure of NME2 with CoA Enabled the Identification of an LCFA-CoA–Resistant NME1/2 Mutant.

**Fig. 3.**
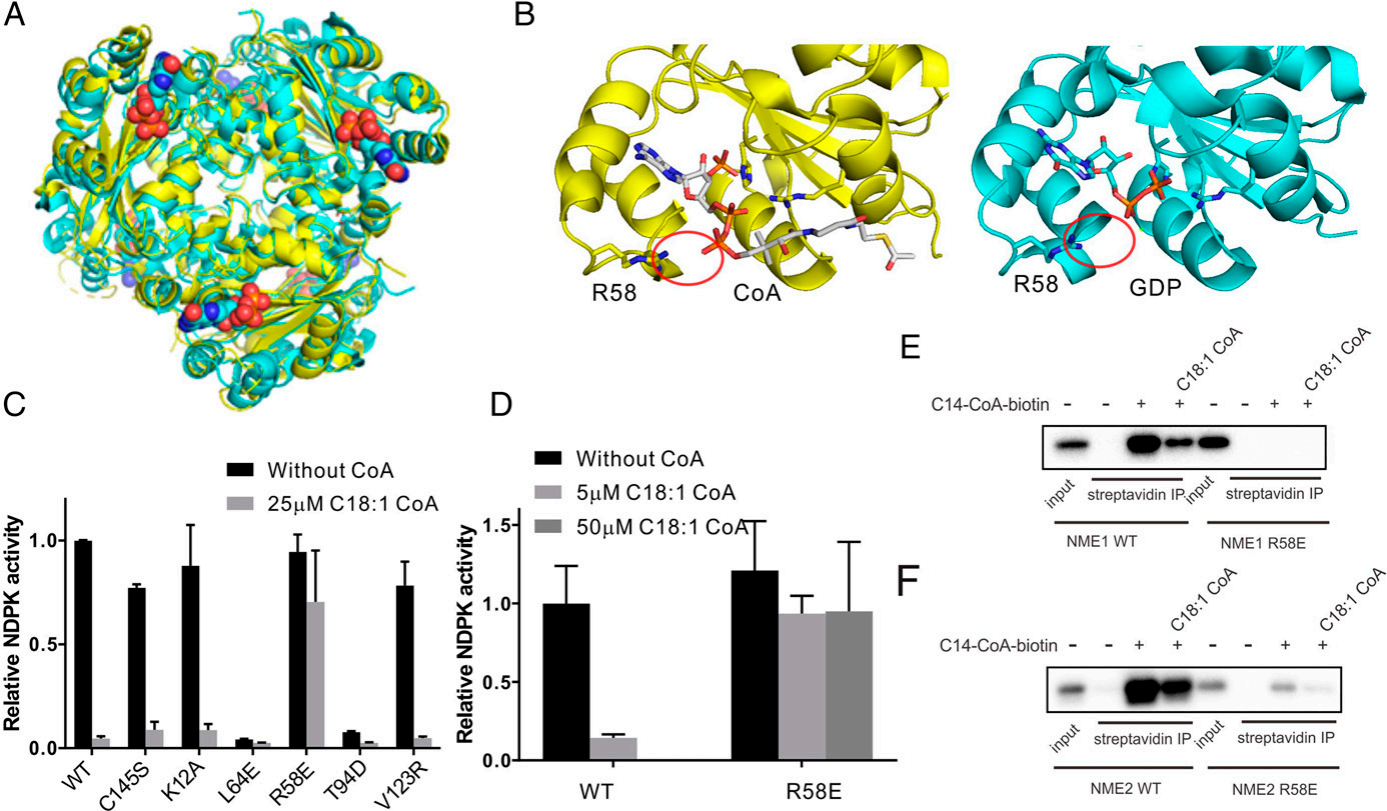
R58 is critical for LCFA-CoA binding on NME proteins. (*A*) Overlay of LCFA-CoA–bound NME2 (yellow) and GDP-bound NME2 (PDB: 3bbf, cyan). The bound GDP is shown in spheres. (*B*) Zoom-in view of LCFA-CoA bound in NME2 active site (yellow) and GDP-bound in NME2 active site (PDB: 3bbf, cyan). The red circles indicate the interaction between R58 and GDP/CoA molecules. (*C*) NDPK activity of different NME2 mutants with or without C18:1 CoA treatment. (*D*) NDPK activity of NME1 R58E with or without C18:1 -CoA treatment. (*E*) Pull-down of NME1 WT and NME1 R58E by C14-CoA-biotin probe in the presence or absence of C18:1 CoA. (*F*) Pull-down of NME2 WT and NME2 R58E by C14-CoA-biotin probe in the presence or absence of C18:1 CoA. For all graphs, the bar heights reflect the mean and the error bars represent SD of independent replicates. IP, immunoprecipitation.

In order to investigate whether the regulation of NME by LCFA-CoA is physiologically or pathophysiologically relevant, we needed to identify an NME mutant that retains catalytic activity but cannot be bound and inhibited by LCFA-CoA. Toward this goal, we tried to cocrystalize NME2 with myristoyl CoA or nonhydrolyzable myristoyl CoA. We were able to obtain crystals and solve the structures. In the structures, the electron density for CoA was clearly visible while that for the fatty acyl chain was not. Careful examination of the structure revealed important information about how LCFA-CoA may bind to NME. When aligning this structure to the reported GDP-bound NME2 structure (Protein Data Bank [PDB]: 3bbf), we found that the overall structures were almost identical. Interestingly, CoA occupied the active site where GDP was bound. However, the residues involved in GDP or CoA binding were different. In particular, R58 made an important salt bridge interaction with CoA, while it did not interact with GDP (Fig. 3*B*).

The structure thus suggests that R58 mutation could potentially eliminate NME inhibition by LCFA-CoA without affecting the normal catalytic activity. We generated a series of NME2 mutant (including R58E) recombinant proteins (*SI Appendix*, Fig. S8). L64E and T94D mutants were catalytically dead, while C145S and V123R mutants showed unaltered NDPK activity and LCFA-CoA–mediated inhibition (Fig. 3*C*). Satisfyingly, the R58E mutation did not affect the NDPK activity of NME2 but conferred resistance to LCFA-CoA inhibition (Fig. 3*C*). The NME1 R58E mutant was also resistant to LCFA-CoA inhibition (Fig. 3*D*). Pull-down with the LCFA-CoA-biotin probe in cell lysate showed that the R58E mutants of NME1 and NME2 were largely defective for LCFA-CoA interaction (Fig. 3 *E* and *F*). Thus, the crystal structure allowed us to identify the partial binding site of LCFA-CoA on NME1/2 and generate mutants of NME1 and NME2 that are resistant to the inhibition by LCFA-CoA.

### LCFA-CoA Regulates Clathrin-Mediated Endocytosis by Inhibiting NME.

**Fig. 4.**
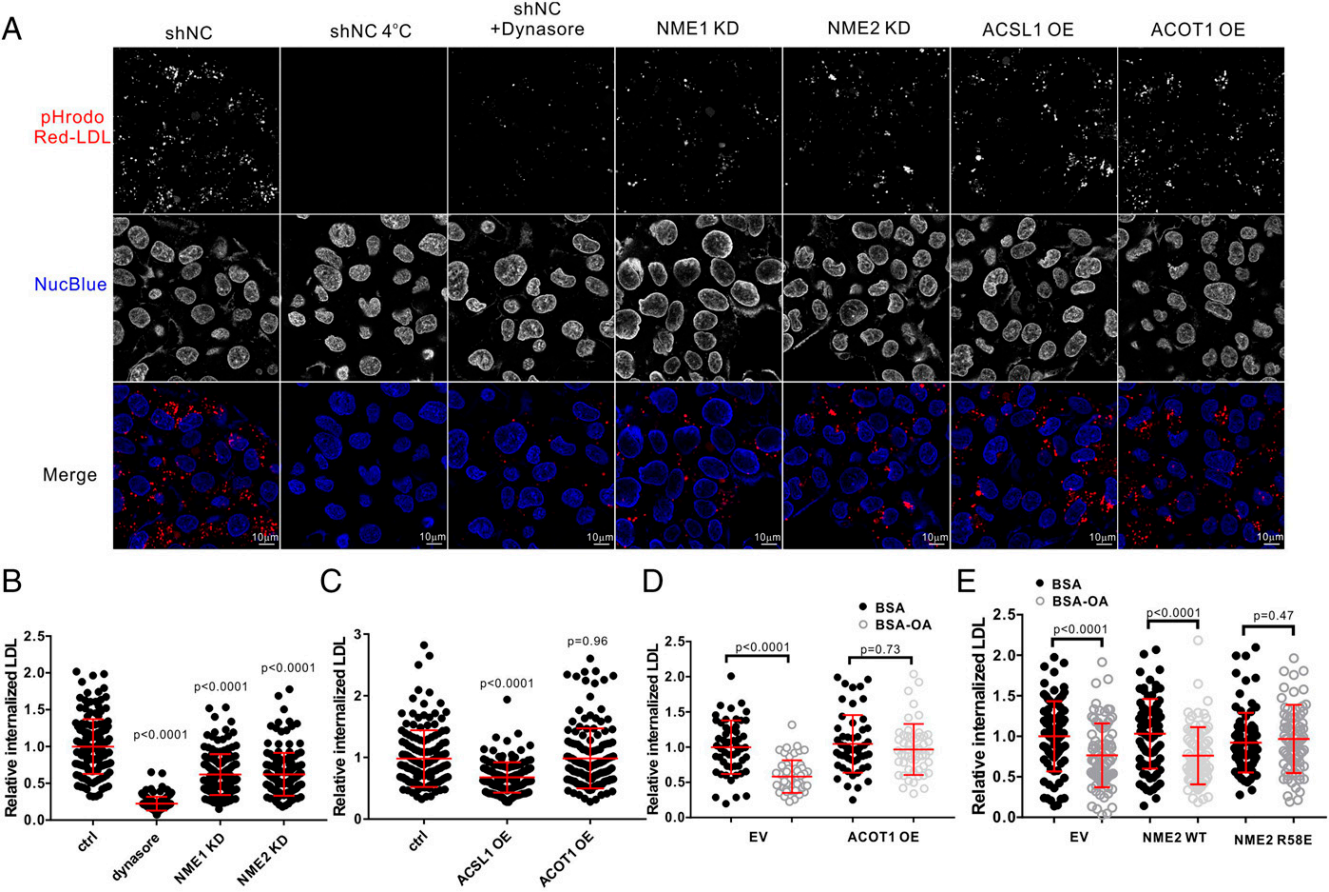
LCFA-CoA regulates clathrin-mediated endocytosis by inhibiting NME proteins in HeLa cells. (*A*) Live cell imaging of pHrodo Red LDL internalization in HeLa cells by confocal microscopy. (*B*–*E*) Relative internalized LDLs were quantified with Cytation 5 imaging station based on intracellular mean fluorescence in cells. (*B*) NME1 or NME2 KD suppressed LDL internalization (*n* = 150 cells). (*C*) The effects of ACSL1 or ACOT1 overexpression on LDL internalization (*n* = 150 cells). (*D*) The effects of ACOT1 overexpression on LDL internalization with oleic acid treatment (*n* = 55 cells). (*E*) The effects of NME2 WT or NME2 R58E overexpression on LDL internalization with oleic acid treatment (*n* = 85 cells). Statistical significance was calculated by unpaired two-tailed Student *t* test. Data shown in the figure are representative of minimally two independent experiments. (Scale bars, 10 µm.) ctrl, control; shNC, negative control shRNA; KD, knockdown; OE, overexpression; OA, oleic acid; EV, empty vector.

We next investigated whether inhibition of NME by LCFA-CoA also happens in cells. To test this, we tried to vary intracellular LCFA-CoA levels and investigated clathrin-mediated endocytosis, in which NME1 and NME2 play important roles by supplying the GTP needed for dynamin-2 (22). If inhibition of NME1/2 by LCFA-CoA happens in cells, increasing LCFA-CoA levels would inhibit endocytosis. We monitored transferrin receptor (TfR), epidermal growth factor receptor (EGFR), and low-density lipoprotein (LDL) receptor internalization in HeLa cells, all of which rely on endocytosis. Fluorescently labeled ligands (Alexa488-Tf, pHrodo Red LDL, and pHrodo Red EGF) were used to track the internalization process by confocal microscopy. NME1 or NME2 knockdown suppressed the internalization of these receptors (Fig. 4*A* and *SI Appendix*, Figs. S7 and S9), which is consistent with previous reports (22). The endocytic defect of transferrin receptor was rescued by expression of wild-type (WT) or R58E mutant NME1 (*SI Appendix*, Fig. S9 *C* and *D*), suggesting that WT and R58E mutant NME1 have similar activity in cells under normal conditions. We quantified the internalized signal in a larger population of cells in each sample with a Cytation 5 imaging station, which can capture images automatically and unbiasedly. Quantification of the results showed that NME knockdown inhibited endocytosis (Fig. 4*B* and *SI Appendix*, Fig. S10). The intracellular availability of LCFA-CoA is tightly controlled by de novo fatty acid synthesis, the activity of acyl-CoA synthetase (ACSL) and acyl-CoA thioesterase (ACOT), the rate of β-oxidation, and finally the concentration of cellular LCFA-CoA–binding proteins (4). ACSL1 overexpression, which increases intracellular LCFA-CoA (23), suppressed the internalization of TfR, EGFR, and LDL receptor (Fig. 4 *A* and *C* and *SI Appendix*, Fig. S11), consistent with our hypothesis that increased LCFA-CoA would inhibit endocytosis via NME1/2. Knockdown of NME1/2 in the background of ACSL1 overexpression did not further suppress endocytosis (*SI Appendix*, Fig. S11 *I* and *J*). Also, overexpression of ACSL1 in the background of NME1/2 knockdown did not further suppress endocytosis (*SI Appendix*, Fig. S11 *K* and *L*). These evidences suggest that ACSL1 overexpression reduces endocytosis by increasing LCFA-CoA concentration and inhibiting NME. ACOT1 overexpression, which decreases intracellular LCFA-CoA levels, did not have a significant effect (Fig. 4 *A* and *C* and *SI Appendix*, Fig. S11). This indicates that NME may not be strongly inhibited at normal low concentrations of LCFA-CoA. The effects of ACSL1 and ACOT1 overexpression on intracellular LCFA-CoA levels were confirmed by liquid chromatography-mass spectrometry analysis in both HeLa cells and MDA-MB-231 cells (*SI Appendix*, Fig. S11 *D* and *F*).

To further confirm that increased LCFA-CoA could inhibit endocytosis, we monitored endocytosis in cells preconditioned with C18:1 oleic acid–bovine serum albumin (BSA) conjugates, which lead to the accumulation of fatty acid metabolism intermediates, including LCFA-CoA (*SI Appendix*, Fig. S11*D*) (24). While saturated palmitic acid and stearic acid are the major fatty acids in human and mouse, in cellular studies, we used C18:1 oleic acid because the saturated fatty acids, especially palmitic acid, are used by cells to palmitoylate thousands of proteins, which would complicate mechanistic analysis and also cause cellular toxicity. As shown in Fig. 2*E*, C18:1 CoA is very potent at inhibiting NME1/2. Consistent with our prediction, 3 h of oleic acid treatment suppressed the internalization of transferrin receptor, LDL receptor, and EGFR (*SI Appendix*, Fig. S12). Interestingly, ACOT1 overexpression, which accelerates LCFA-CoA hydrolysis, relieved this inhibition (Fig. 4*D* and *SI Appendix*, Fig. S13*A*). This suggests that LCFA-CoA accumulation contributed to oleic acid–induced suppression of endocytosis.

To confirm that the inhibition of endocytosis by oleic acid was through NME, we ectopically expressed either WT NME2 or the R58E mutant and examined the effect of oleic acid treatment on endocytosis. The NME2 R58E mutant was not inhibited by LCFA-CoA; thus, we expected that it would protect endocytosis from the inhibitory effect of oleic acid. LDL receptor and transferrin receptor internalization in NME2 WT–expressing cells was sensitive to oleic acid treatment, while in NME2 R58E–expressing cells it was insensitive to oleic acid treatment (Fig. 4*E* and *SI Appendix*, Fig. S13*B*). We further checked transferrin internalization in MDA-MB-231 cells stably expressing NME1 WT or NME1 R58E and mouse embryonic fibroblasts cells generated from transgenic mice expressing either NME1 WT or R58E and obtained consistent results (*SI Appendix*, Fig. S13 *D*–*F*). The data strongly support that oleic acid–induced endocytosis suppression is due to the inhibition of NME proteins by LCFA-CoA. Altogether, these results support that LCFA-CoA suppresses endocytosis through inhibiting NME.

### Inhibition of NME by LCFA-CoA Supports Breast Cancer Metastasis.

Although not completely understood, NME1 has been well-documented to regulate tumor metastasis (8, 25). On the other hand, the fatty acid transporter CD36 has been found to promote tumor metastasis under high nutrient conditions in various cancer types, such as oral cancer, breast cancer, and melanoma (26). Given that LCFA-CoA could inhibit NME, we hypothesized that NME1 inhibition by LCFA-CoA could provide a key molecular link between fatty acid uptake/metabolism and breast cancer metastasis.

**Fig. 5.**
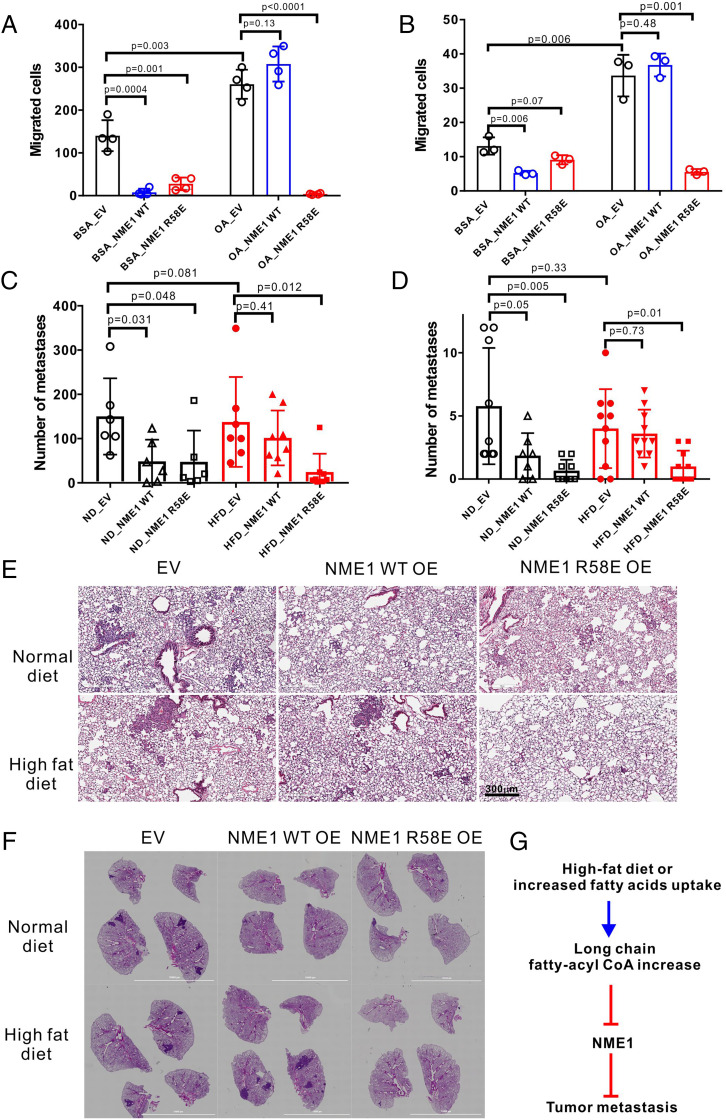
Inhibition of NME by LCFA-CoA contributes to breast cancer metastasis in mouse exposed to an HFD. Quantification of migrated cells in the Boyden chamber migration assay for (*A*) MDA-MB-231 cells and (*B*) E0771 cells. Quantification of the metastasis per mouse for (*C*) MDA-MB-231 cells in NSG mice and (*D*) E0771 cells in C57BL/6J mice. The representative images of the lung sections from each group from (*E*) NSG mice and (*F*) C57BL/6J mice. (*G*) A model for the regulation of LCFA-CoA on tumor metastasis via NME1/2 inhibition. The elevated LCFA-CoA, which could be due to exposure to an HFD or increased fatty acid uptake, inhibits NME1, compromising the metastasis suppression function of NME1. Statistical significance was calculated by unpaired two-tailed Student *t* test. For all graphs, the middle lines reflect the mean and the error bars represent SD of independent biological replicates. EV, empty vector; OA, oleic acid; OE, overexpression.

NME1 protein level is down-regulated in metastatic breast cancer cell lines compared to nontransformed or nonmetastatic cell lines (27) (*SI Appendix*, Fig. S14*G*). Overexpression of NME1, but not its highly related isoform NME2, has been reported to suppress cell migration in multiple highly invasive breast cancer cell lines (8, 14). In a Boyden chamber migration assay, both WT and R58E mutant NME1 expression significantly suppressed cell migration under serum-starved conditions in multiple breast cancer cell lines (Fig. 5 *A* and *B* and *SI Appendix*, Fig. S14*A*). Consistent with previous reports, treatment of control cells with BSA–oleic acid conjugates promoted cell migration (Fig. 5 *A* and *B* and *SI Appendix*, Fig. S14*A*) (28–30). Interestingly, upon oleic acid treatment, WT NME1 expression failed to suppress cell migration, whereas R58E mutant expression continued to suppress cell migration as in the absence of BSA–oleic acid (Fig. 5 *A* and *B* and *SI Appendix*, Fig. S14*A*). We also performed invasion assays using a Boyden chamber coated with Matrigel with MDA-MB-231 cells and obtained similar results (*SI Appendix*, Fig. S14*B*). We noted that the forced expression levels of WT and R58E mutant of NME1 were not higher than the levels of NME1 in nonmetastatic breast cancer cell lines (*SI Appendix*, Fig. S14*G*). These results strongly support that NME1-dependent metastasis suppression is compromised by LCFA-CoA inhibition.

To further confirm that NME1 inhibition by LCFA-CoA regulates breast cancer metastasis in vivo, we studied spontaneous lung metastasis of breast cancer in both immunodeficient and immunocompetent orthotopic mouse models: 1) human breast cancer cell MDA-MB-231 in NSG mice and 2) mouse breast cancer cell E0771 in C57BL/6J mice. Considering that the metastasis-promoting effect of fatty acid uptake may particularly rely on dietary lipids (26), we included groups of mice on an HFD. NSG or C57BL/6J female mice were divided randomly into two groups and fed with either a HFD or a normal diet (ND). The mice on an HFD gained more body weight than those on a ND after 6 wk, and the body weight became relatively stable after that (*SI Appendix*, Fig. S15 *A* and *B*). MDA-MB-231 or E0771 cells stably expressing empty vector (EV) control, WT NME1, or R58E mutant NME1 were then injected orthotopically into the mammary fat pad of the mice. Nine wk for MDA-MB-231 cells and 4 wk for E0771 cells after the injection, the mice were euthanized, and the primary tumor and lung metastasis were analyzed. We also randomly took four tumor samples expressing control empty vector from mice on an HFD or ND and checked the intracellular LCFA-CoA levels using targeted metabolomics analysis. As anticipated, we observed an increase of major intracellular LCFA-CoA species in samples from HFD-fed mice (*SI Appendix*, Fig. S15). Exposure to HFD promoted primary tumor growth, although the effect in tumors expressing the R58E mutant in NSG mice was not statistically significant (*P* = 0.24). NME1 WT or R58E overexpression did not significantly affect primary tumor growth, as expected (*SI Appendix*, Fig. S16).

HFD feeding did not further promote lung metastasis of control MDA-MB-231 or E0771 tumors in our experimental conditions. This could be because both cell lines are highly invasive and have a lower level of NME1 compared to low-metastatic carcinoma cells (8). On a ND, WT and R58E NME1 overexpression suppressed lung metastasis potently (Fig. 5 *C*–*F*). Interestingly, when exposed to an HFD, the metastasis-suppressing effect of NME1 WT was abolished. In contrast, metastasis was still strongly suppressed by NME1 R58E when the mice were fed an HFD (Fig. 5 *C*–*F*). This result suggests that the inhibition of NME1 by LCFA-CoA contributed to breast cancer metastasis in mice exposed to an HFD. Thus, our data together support a model that NME1 inhibition by LCFA-CoA is important for the metastasis of breast cancer exposed to oleic acid in vitro or an HFD in vivo.

## Discussion

Here, we used a quantitative chemical proteomic approach to systematically identify proteins that can bind to LCFA-CoA. In addition to identifying many proteins that are known to utilize LCFA-CoA as substrates, we also identified proteins that do not use LCFA-CoA as substrates. These proteins are thus likely regulated by LCFA-CoA. In further biochemical studies, we focused on two enzymes, NME1/2, which were not previously known to utilize or bind to LCFA-CoA. Interestingly, LCFA-CoA potently inhibits NME1/2 in vitro. Increasing LCFA-CoA concentrations in cells inhibits clathrin-mediated endocytosis, a process that is promoted by the activity of NME1/2. Our study thus revealed a role of LCFA-CoA in regulating the activity of nucleotide metabolic enzymes.

Fatty acid metabolism is reported to be important for cancer metastasis (6, 31, 32). The discovery that the fatty acid transporter CD36 plays indispensable roles for cancer metastasis (26) further highlights the importance of fatty acid metabolism in cancer metastasis. However, the mechanisms via which fatty acid metabolism affects metastasis is not clear. We hypothesized that LCFA-CoA, as an important metabolite in lipid metabolism, may regulate certain proteins that promote cancer metastasis. NME1 is known to be a metastasis suppressor (7, 8). Our study here showed that LCFA-CoA, by inhibiting NME1, contributes to breast cancer metastasis in mice exposed to an HFD. This is a previously unknown molecular mechanism that could explain in part how fatty acid uptake affects tumor metastasis. On the other hand, reexpression of NME1 in invasive cancer cells has been thought to be a promising strategy for antimetastatic therapy (33, 34); our results indicate that the suppression of NME1 by LCFA-CoA needs to be considered in future clinical practice.

NME1 has multiple roles in cancer metastasis involving epithelial-mesenchymal transition (35), matrix proteolysis, and proinvasive signaling (36). This makes NME1 a major upstream regulator of the metastatic signaling cascade (8). On the other hand, LCFA-CoAs are the immediate active form of fatty acids, important nutrients for cancer cells. The interaction between NME1 and LCFA-CoA may represent a way for cancer cells to sense the dynamic changes of intracellular LCFA-CoA abundance, which helps the cancer cells to coordinate proliferation and invasion.

An intriguing question is why this mechanism evolved to regulate NME1/2 activity. A possible hypothesis could be gleaned from our data showing that LDL endocytosis is inhibited by LCFA-CoA via NME1/2. LDL endocytosis is the mechanism for human cells to uptake lipids from the circulation (37). Perhaps when cellular lipid abundance is high, indicated by rising LCFA-CoA concentrations, cells benefit from decreasing LDL endocytosis by LCFA-CoA–mediated NME inhibition. Unfortunately, this same mechanism that helps prevent lipid overload under normal homeostatic conditions is utilized by tumors to promote metastasis.

Our study also demonstrated the utility of the quantitative chemical proteomic approach for discovering the regulatory functions of small molecule metabolites. The quantitative chemical proteomic profiling identified not only proteins that are known to utilize LCFA-CoA but also proteins that are regulated by LCFA-CoA through noncovalent binding. The quantitative information also helps focus our attention on high-confident hits, facilitating the downstream biochemical studies. We believe this could represent a facile general approach to study the regulation of protein function by many other small molecule metabolites.

## Methods

Detailed methods, including the preparation of C14-CoA-biotin, the chemical proteomic approach, the cellular endocytosis assay, breast cancer cell migration and invasion, and mouse models, can be found in the supporting information. Studies involving mice followed protocols approved by the Institutional Animal Care and Use Committee of Cornell University.

## Supplementary Material

Supplementary File

Supplementary File

## Data Availability

X-ray crystal structure data of NME2 bound to myristoyl-CoA have been deposited into the PDB (accession no. 7KPF, https://www.rcsb.org/structure/7KPF).
