# Supplementary material for: Long-chain fatty acyl coenzyme A inhibits NME1/2 and regulates cancer metastasis
Source: Proc Natl Acad Sci U S A. 2022 Mar 8;119(11):e2117013119. doi: 10.1073/pnas.2117013119 (PMC8931372; doi:10.1073/pnas.2117013119)
Supplement: Supplementary File [file pnas.2117013119.sapp.pdf]

Supplementary Information

Long-Chain Fatty Acyl Coenzyme A inhibits NME1/2 and regulates cancer metastasis under high-fat diet

Shuai Zhang,[1,2] Ornella D. Nelson,[2] Ian R. Price,[2] Chengliang Zhu,[2] Xuan Lu,[2] Irma R. Fernandez,[2,3] Robert S. Weiss,[3] Hening Lin[1,2,*]

[1]Howard Hughes Medical Institute; Department of Chemistry and Chemical Biology, Cornell University, Ithaca, NY 14853, USA

[2]Department of Chemistry and Chemical Biology, Cornell University, Ithaca, NY 14853, USA

[3]Department of Biomedical Sciences, Cornell University, Ithaca, NY 14853, USA

*To whom correspondence may be addressed. E-mail: hl379@cornell.edu

Supplementary Methods

Synthesis of the C14-CoA-Biotin probe. The synthesis was done in three steps from 15-hexadecy-1-oic acid (**Alk14**), which was prepared following a known procedure.[1, 2]

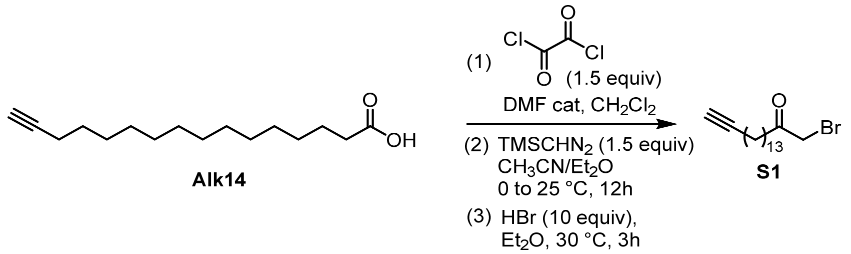

To a flame-dried 50 mL round bottom flask equipped with a stir bar was added **Alk14** (100 mg, 0.4 mmol, 1.0 eq.). After the flask was evacuated and backfilled with N_2 twice, anhydrous CH_2Cl_2 (20 mL) were added via a syringe. One drop of DMF was added and the mixture was cooled down to 0 °C. Oxalyl chloride (51 µL, 0.6 mmol, 1.5 eq.) was added dropwise. The reaction mixture was moved to room temperature, stirred for additional 3 h and concentrated under reduced pressure. The residue was

dissolved in CH_3CN (1 mL) and cooled down to 0 °C. (Trimethylsilyl)diazomethane (2.0 M Et_2O solution) (0.3 mL, 1.5 eq.) was added slowly and the mixture was allowed to gradually warm up to room temperature and vigorously stirred for 16 h under N_2. After the reaction was completed (monitored by TLC), the solvent was removed under reduced pressure and the residue was dissolved in Et_2O (15 mL). Hydrogen bromide (37% in H_2O, 10.0 eq.) was added slowly and the mixture was refluxed for 3 h. Saturated $NaHCO_3$ solution (15 mL) was added to quench the reaction. The organic phase was separated from the aqueous phase, and the aqueous phase was extracted with EtOAc (20 mL × 2). The combined organic phase was dried over Na_2SO_4. After concentration *in vacuo*, the residue was subsequently purified through column chromatography (hexanes/Et_2O: from 15:1 to 9:1) to afford **S1** as a colorless semi-solid (90 mg, 69% yield from **Alk14**).

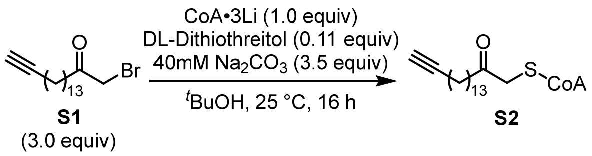

To a 25 mL round bottom flask equipped with a stir bar was added coenzyme A trilithium salt (45 mg, 0.056 mmol, 1.0 eq.) and DL-dithiothreitol (1.0 mg, 0.0065 mmol, 0.11 eq.). After the flask was evacuated and backfilled with N_2 twice, Na_2CO_3 aqueous solution (0.04 M, 5 mL) were added via a syringe. The mixture was stirred for 30 min at room temperature and then a solution of **S1** (56 mg, 0.17 mmol, 3.0 eq.) in tert-butanol (7 mL) was added. The reaction mixture was stirred for 16 h under room temperature. The solvent was removed under reduced pressure and the residue was

dissolved in water (15 mL). The mixture was filtered through a pad of Celite and the filtrate was frozen and lyophilized to remove water. The obtained crude white solid was directly used in the next step without further purification.

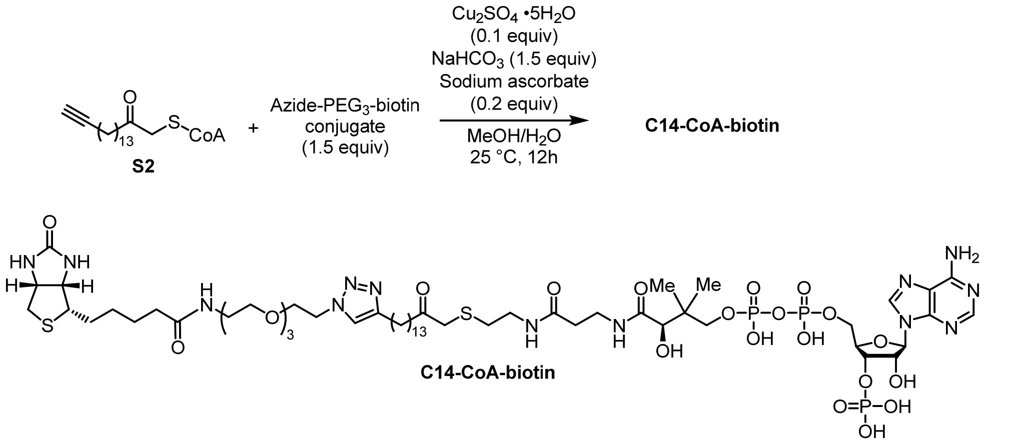

To a 2-dram vial equipped with a stir bar was added the crude **S2** (5.6 μmol, 1.0 eq.). After the vial was evacuated and backfilled with N_2 twice, degassed water (1.0 mL) and MeOH (1.0 mL) were added via a syringe. Subsequently, $NaHCO_3$ (100 μL, 8.4 mM stock solution), $CuSO_4$•$5H_2O$ (100 μL, 0.56 mM stock solution), sodium ascorbate (100 μL, 1.12 mM stock solution) and azide-PEG_3-biotin conjugate (170 μL, 50 mM stock solution) were added and the reaction mixture was stirred at room temperature for 12 h and monitored by LC-MS. After concentration *in vacuo*, the residue was redissolved in H_2O and subsequently purified through reverse phase prep HPLC with 0.1% trifluoroacetic acid in water (solvent A) and 0.1% trifluoroacetic in acetonitrile (solvent B) (0% solvent B for 10 min, 0–30% solvent B in 30 min, 30% B for 10 min, flow rate 0.5 mL/min) , t_r = 8.36 min.. After Lyophilization, **C14-CoA-biotin** was obtained as a white solid. LR-MS (ESI, *m/z*): calcd for $C_{56}H_{96}N_{13}O_{22}P_3S_2^{2+}$ (M + $2H^+$), 730.77,

found 730.96; calcd for $C_{56}H_{96}N_{13}O_{22}P_3S_2^{3+}$ (M + $3H^+$), 487.51, found 487.49.

Reagents. Antibodies for IDH2 (#12652), ACSL1 (#9189), NME1 (#3345), and GLUD1/2 (#12793) were purchased from Cell Signaling Technology. Antibodies for NME1 (SC-514515), NME2 (SC-100400), KIF4 (sc-365144), ACBP (sc-376853) and Dynamin Inhibitor I, Dynasore (sc-202592) were purchased Santa Cruz Biotechnology. ACOT1 (SAB4300908) antibody, antiFLAG affinity gels (#A2220) and FLAG antibody (#A8592) were purchased from Sigma. Human NME1 shRNAs (#1, TRCN0000010055, #2, TRCN0000010061, #3, TRCN0000194801, #3 targets the 3'UTR) and human NME2 shRNAs (#1, TRCN0000379743, #2, TRCN0000195380) were purchased from Sigma. Protease inhibitor cocktail, phosphatase inhibitor cocktail 3, crystal violet, [$^{13}C_6$, $^{15}N_2$]-L-lysine, [$^{13}C_6$, $^{15}N_4$]-L-arginine, L-lysine, L-arginine, C12:0 CoA lithium salt, C14:0 CoA lithium salt, C16:0 CoA lithium salt, C16:1 CoA lithium salt, C18:0 CoA lithium salt, C18:1 CoA lithium salt were purchased from Sigma. n-Heptadecanoyl coenzyme A lithium salt (C17:0 CoA lithium salt) was purchased from Cayman Chemical. FuGENE 6 transfection reagent and sequencing grade modified trypsin were purchased from Promega. ECL and ECL plus Western blotting detection reagent, pHrodo™ Red-LDL, pHrodo™ Red Epidermal Growth Factor (EGF), Transferrin-Alexa Fluor™ 488 Conjugate were purchased from Thermo Fisher. ACBP recombinant proteins (#Pro-832) was purchased from Prospec.

Cell Culture. Human embryonic kidney (HEK) 293T cells, Hela cells, E0771, MDA-MB-231 and MDA-MB-468 breast cancer cells were cultured in Dulbecco's modified

Eagle medium (DMEM) medium (ThermoFisher) with 10% FBS (ThermoFisher).

Cloning, Transfection, and Transduction. Human NME1, NME2, ACSL1, and ACOT1 were inserted into pCMV4a (C terminal FLAG tag) and pCDH-CMV-MCS-EF1-Puro vectors. All transient transfections were performed using FuGENE 6 transfection reagent according to the manufacturer's protocol. NME1, NME2, ACOT1, ACSL1, NME1 shRNA and NME2 shRNA lentiviruses were generated by co-transfection of NME1/NME2/ACSL1/ACOT1 in pCDH vector or NME1/NME2 shRNA in pLKO.1 vector, pCMV-dR8.2, and pMD2.G into HEK293T cells. To obtain the stable overexpression or knockdown Hela, E0771, MDA-MB-468 and MDA-MB-231 cells, cells were treated with 2 μg/mL of puromycin 48 h after lentiviral infection.

SILAC and Nano LC−MS/MS Analysis. "Heavy" HEK293T cells were cultured in DMEM with [$^{13}C_6$, $^{15}N_2$]-L-lysine, [$^{13}C_6$, $^{15}N_4$]-L-arginine, and 10% dialyzed FBS for 5 generations. "Light" HEK293T cells were cultured in DMEM with normal L-lysine, L-arginine, and 10% dialyzed FBS for 5 generations. The "heavy" and "light" cells were collected and lysed in 1% NP40 lysis buffer separately, according to the workflow described above. Protein input of 8 mg for each "heavy" and "light" total lysate was treated with either DMSO or 25 μM of C14-CoA-biotin probe at 4°C for 1h. Then both "heavy" and "light" samples were subjected for streptavidin immunoprecipitation separately. After washing the affinity gel three times with NP40 washing buffer,"heavy" and "light" samples were mixed and washed two more times with NP40 washing buffer. Then on-beads trypsin digestion was conducted and the digested peptide solutions were collected and sent for LC−MS/MS analysis (LTQ-Orbitrap Elite mass spectrometer

coupled with nanoLC) as previous reported[3].

Construct design and purification. The NME2 gene (1-152) was cloned into a His-SUMO-pET28a vector. BL-21 cells were grown up in 1.5x LB at 30° C until an OD of 0.6. Expression was induced by 0.1 mM IPTG and the protein was expressed for 18 h at 18° C. Cell pellets were resuspended in 25 mM Tris pH 8, 400 mM NaCl, 0.5 mM PMSF. Cells were lysed by sonication and the protein was isolated from the cleared lysate by Co-NTA resin (Qiagen). The tag was removed by cleavage with ULP1 protease concurrent with dialysis, followed by reverse Co-NTA chromatography to remove the His-SUMO. The flow-through was concentrated and purified by size exclusion on a Superdex 200 26/600 column. The cleaved NME2 runs as a hexamer on size exclusion chromatography. To allow solubility of the myristoyl-CoA in the crystallization solution, a buffer of 30 mM HEPES pH 7.4, 1 mM EDTA, 1 mM DTT was used. The protein was concentrated to 34 mg/mL (2 mM) and used fresh or flash frozen and stored at -80° C.

NDPK activity assay. NDPK assay was performed in the kinase buffer (50mM triethanolamine, 150mM NaCl, 2mM MgCl2, 1mM EDTA and 1mM DTT) with 50nM of purified NME proteins, 500uM GDP and 1mM ATP at room temperature for 5 min. The reaction is quenched by adding formic acid (0.28M final concentration). The reaction solution is then diluted 3 folds with pure water and analyzed by reversed phase ion-pair HPLC method[4]. Briefly, Separation of nucleotides by reversed phase ion-pair chromatography with analytical HPLC (Shimadzu UFLC). Chromatographic conditions: column, Kinetex 5 µm EVO C18 100 Å, 150 × 4.6 mm from Phenomenex;

mobile phase, Buffer A (5 mM tetrabutylammonium phosphate, 4% acetonitrile and 50 mM potassium dihydrogen phosphate, pH 6.0), Buffer B (acetonitrile), a gradient from 0-30% buffer B was used over 45 min; flow rate, 0.5 ml/min; detection, UV at 254 nm. The conversion rate of GDP to GTP is used as indicator for NDPK activity. All NDPK activities were measured based on n = 3 independent experimental replicates.

Crystallization and Structure Solution. For crystallization, the 2 mM NME2 protein was mixed at a 1:1.1 ratio with 2 mM myristoyl-CoA in water and incubated on ice for 15 min before sparse matrix screening. NME2 produced diffracting crystals from 6 different crystal forms. The best result, presented here, was crystallized by favor diffusion at 20° C with a well solution of 10% PEG-4000, 100 mM $MgCl_2$, 100 mM HEPES pH 7.5 and a protein-to-well drop ratio of 1:1. Structures resulting from other conditions produced similar structural results. A non-hydrolyzable myristoyl-CoA analog was also tried, with similar results. Crystals were flash frozen after cryoprotection in well solution plus a final additional 4% PEG and 20% glycerol, added over 3 incremental steps.

Diffraction images were collected at the Advanced Photon Source, NE-CAT beamline. The data was processed by XDS[5] and Aimless[6], and the structure was solved by molecular replacement with Phaser[7] as part of the NECAT beamline's RAPD pipeline. PDB entry 3BBF was used as the model.[8] Building and ligand modeling were performed in Coot.[9] Refinement and validation were performed with the Phenix suite.[10]

Confocal or Cytation 5 Imaging for endocytosis. Cells were starved in DMEM with BSA or BSA-oleic acid conjugates for 3 h. After washing twice with ice cold PBS, the

cells were incubated for 30 min in ice-cold DMEM containing 5 µg/mL Alexa488-conjugated human Tf, 5 µg/mL pHrodo™ Red-LDL, or 5 µg/mL pHrodo™ Red-EGF. After washing with ice-cold DMEM, cells were incubated in DMEM at 37°C for the indicated times (typically 15 min for Tf, 30 min for LDL and EGFR) and quickly cooled on ice. For pHrodo™ Red-LDL and pHrodo™ Red-EGF treated cells, images were taken in ice-cold imaging solution with NucBlue™ Live ReadyProbes™ Reagent. For Alexa488-conjugated human Tf, cells were rinsed with cold PBS twice, acid-washed in ice-cold stripping medium (50 mM glycine, 100 mM NaCl, pH 3.0)

for 2 min to remove surface-bound Tf, wash with cold PBS twice again and fixed with 4% paraformaldehyde (in PBS) for 15 min. The fixed cells were washed twice with PBS, applied with one drop of antifade reagent (with DAPI) and then imaged with Zeiss LSM880 inverted confocal microscopy or Cytation 5 imaging station. For imaging on Cytation 5, the images were captured in experiment mode which automatically captures images with the same settings for all the samples in each experiment. The NucBlue/DAPI signal was used to autofocus when capture the images. The intracellular analysis was performed using Gen5. Cells were located with the NucBlue/DAPI signal and the internalized fluorescence signals. Backgrounds were subtracted in each image and the intracellular mean fluorescence in each cell were used for the quantification.

Targeted metabolomic analysis of LCFA-CoAs in cell. MDA-MB-231 or Hela cell cultures (~2×10^6 cells) were placed on ice, the medium was aspirated, and adherent cells were gently washed twice with cold PBS. The cells were rapidly scraped and collected. Small fraction of the cell suspension was saved for cell quantitation by

counting. For tumor samples, frozen tumor pieces were ground to a fine homogenous powder using a mortar and pestle cooled with liquid nitrogen. 50 mg of tumor powder was weighed into sample tubes.

The samples were collected and resuspended in a cold solution of 80% MeOH, vigorously vortexed for 15s and immediately frozen in liquid nitrogen. The internal standard, n-Heptadecanoyl coenzyme A (C17:0 CoA), was added to the frozen cells at 20pmol/10^6 cells or 2pmol/10mg tissue right before thawing the cells on ice. The samples were then thawed on ice, sonicated for 30 s and incubated for 15 min on ice for the metabolite extraction. After centrifuged at 21,000 g for 15 min, the supernatants were collected and freeze dried. All cell samples were made in three biological replicates and tumor samples were mad in 4 biological replicates.

Dried cell extract containing the internal standard C17:0 CoA was reconstituted right before LC-MS analysis with 50 μl of 50 mM ammonium acetate (pH 6.8) in aqueous solution containing 20% (vol/vol) acetonitrile. Sample solutions were then centrifuged at 16,000 × g at 4 °C for 5 min and the supernatant was transferred to LC vials.

Shimadzu Exion UHPLC system was coupled to a SciexX500B QTOF mass spectrometer for the analysis of LCFA-CoA in the samples. A reverse-phase liquid chromatography method was used for separating the analytes using Luna C18 column (100 × 2.0 mm i.d., 3 μm; Phenomenex) and then samples were injected to mass spectrometer. Solvent A was 10 mM ammonium acetate (pH 8.5) in water, and solvent B was acetonitrile. The gradient used for the method was: 0 min, 5% solvent B; 3 min,

5% solvent B; 8 min, 15% solvent B; 8.5 min, 55% solvent B; 14 min, 95% solvent B; 14.5 min, 95% solvent B; and 20 min, 5% solvent B.

The Sciex X500B QTOF mass spectrometer with an ESI source was operated in the negative ion mode for this analysis. The electrospray voltage was set at 4.5 kV and the temperature of the heated capillary was set at 350°C. It was operated under the Ion Source gas 1 and 2 at 30 psi, Curtain gas at 30 (arbitrary unit), CAD gas at 7 (arbitrary unit). The declustering potential (DP) was set to -40V with accumulation time of 0.05s. The MS full scan measurement was done from m/z 100 to m/z 1000 in profile mode followed by multiple reaction monitor (MRM) high-resolution scan acquired from 0 min to 19 min at one collision energies for each analyte. The ion transitions were monitored for each LCFA-CoA in MRM mode. Collision energy values were optimized to 50-70% for these transitions. The raw data were acquired and processed using Sciex OS 1.7 software and the quantitation ratio between each LCFA-CoA/ C17:0 CoA was calculated for each sample by integrating the peak areas of each analyte using MRM transitions in the same software.

Mouse Embryonic Fibroblasts (MEFs) generation. The CRISPR edited $NME1^{R58E}$ mice were generated by Cornell Stem Cell and Transgenic Core Facility. FVB/N female mice were crossed to albino B6 male mice to produce FVBxB62J F1 hybrid embryos. The genotyping was performed with tail tissues and confirmed by sequencing (Genotyping primers: Forward: GTAACAGGCTTCAGAGGACC, Reverse: CAGACTCACCATAGCAACCAC). Embryos from $NME1^{WT}$ or $NME1^{R58E}$ mice were isolated between E13.5 and E15.5. After the heads and most of the internal organs were

removed, the embryos were minced, broken up by pipetting and then seeded into 10-cm cell culture dishes in complete MEF media. The heads were saved for genotyping. The MEF cells were then subjected to large T infection and selected with 2μg/ml puromycin.

Migration and Invasion assay. The migration assay was conducted with Corning Transwell with 8.0 μm PET membrane in two 24-well plates. MDA-MB-231, MDA-MB-468 or E0771 cells (expressing empty vector, NME wt, or R58E mutant) were pre-conditioned with BSA or BSA-oleic acid conjugates (250 μM final oleic acid concentration) for 3 h in serum-free DMEM. The cells were collected and resuspended in serum-free DMEM with BSA or BSA-oleic acid conjugates (250 μM final oleic acid concentration) at indicated seeding: 5×10^4 cells for MDA-MB-231, 2×10^5 cells for MDA-MB-468, 2×10^5 cells for E0771. DMEM with 10% FBS and BSA or BSA-oleic acid conjugates (250uM final concentration of oleic acid) were added to the wells of the Falcon TC Companion Plate (0.75ml). Sterile forceps were used to transfer the chambers and control inserts to the wells. Immediately, 0.5 ml of cell suspension were added to the chambers. The chambers were incubated for 24 hours in a humidified tissue culture incubator at 37°C, 5% CO_2 atmosphere. Cells on the upper surface of the filter were detached with a cotton swab and cells on the lower surface of the filters were fixed with ice-cold methanol for 10 min and stained with 0.1% crystal violet for 15 min. The cells were then rinsed with distilled water, photographed and counted. The average number of migrated cells in 3 random microscopic fields was taken as the migrated cells for each sample. four biological replicates of each sample were used for

quantification.

The invasion assay was conducted with Corning® BioCoat™ Matrigel® Invasion Chambers with 8.0 μm PET membrane in two 24-well Plates. MDA-MB-231 cells (expressing empty vector, NME wt, or R58E mutant) were pre-conditioned with BSA or BSA-oleic acid conjugates (250 μM final oleic acid concentration) for 3 h in serum-free DMEM. The cells were collected and resuspended in serum-free DMEM with BSA or BSA-oleic acid conjugates (250 μM final oleic acid concentration) at 6×10^4 cells/ml. DMEM with 10% FBS and BSA or BSA-oleic acid conjugates (250uM final concentration of oleic acid) were added to the wells of the Falcon TC Companion Plate (0.75ml). Sterile forceps were used to transfer the chambers and control inserts to the wells. Immediately, 0.5 ml of 231 cell suspension ($3\text{x}10^4$ cells) were added to the 24-well chambers. The chambers were incubated for 24 hours in a humidified tissue culture incubator at 37°C, 5% CO_2 atmosphere. Cells on the upper surface of the filter were detached with a cotton swab and cells on the lower surface of the filters were fixed with ice-cold methanol for 10 min and stained with 0.1% crystal violet for 15 min. The cells were then rinsed with distilled water, photographed and counted. The average number of migrated cells in 4 random microscopic fields was taken as the relative migrated cells for each sample. Six biological replicates of each sample were used for quantification.

Spontaneous metastasis mouse model. 1) NSG female mice at age of 4 weeks old were collected and assigned into 6 groups:

Group 1 (n=6): normal diet, with MDA-MB-231 cells bearing empty vector control

Group 2 (n=6): normal diet, with MDA-MB-231 cells overexpressing NME1_WT

Group 3 (n=6): normal diet, with MDA-MB-231 cells overexpressing NME1_R58E

Group 4 (n=7): high-fat diet, with MDA-MB-231 cells bearing empty vector control

Group 5 (n=8): high-fat diet, with MDA-MB-231 cells overexpressing NME1_WT

Group 6 (n=8): high-fat diet, with MDA-MB-231 cells overexpressing NME1_R58E

2) C57BL/6J female mice at age of 6 weeks old were collected and assigned into 6 groups:

Group 1 (n=9): normal diet, with E0771 cells bearing empty vector control

Group 2 (n=7): normal diet, with E0771cells overexpressing NME1_WT

Group 3 (n=9): normal diet, with E0771 cells overexpressing NME1_R58E

Group 4 (n=10): high-fat diet, with E0771 cells bearing empty vector control

Group 5 (n=10): high-fat diet, with E0771 cells overexpressing NME1_WT

Group 6 (n=10): high-fat diet, with E0771 cells overexpressing NME1_R58E

The high-fat diet (diet D12492; Research Diets Inc., New Brunswick, New Jersey, USA), which approximately 60% of calories are from fat, is widely used for diet-induced obesity research. The control mice are fed a control normal diet (diet D12450J; Research Diets Inc., New Brunswick, New Jersey, USA), which 10% of calories are from fat (Matching Sucrose to D12492). These mice will be fed with the corresponding diets for 6 weeks to induce obesity in the high-fat diet groups. During the 6-week period, mice body weight was measured weekly. Then, 5×10^5 MDA-MB-231 cells or 2×10^5 E0771 cells were injected orthotopically into mammary gland. All cell lines were collected using Versene (EDTA, Invitrogen) and mixed on ice with PBS and phenol-

red free Cultrex basementmembrane extract (BME, type 3, Trevigen) to a concentration about 15 mg/ml of BME. Mice were injected bilaterally with tumor cells into each mammary gland under anesthetization with isoflurane. Following the injection, mice were permitted to recover, and were monitored for tumor development. The NSG mice were continued on their diets for 9-10 weeks while C57BL/6J were continued on their diets for 4 weeks. During this period, body weight was measured every week and behavior were evaluated. Then, all mice will be euthanized and sacrificed. The lung and primary tumor tissues were obtained from mice. Primary tumor tissues were weighed, and the lung tissues were fixed in formalin, embedded in paraffin, processed, and stained with Hematoxylin and Eosin (H&E) for examination using Aperio CS2 (Leica) and Cytation 5. Two or three sections of lungs at different depth was processed and stained for each sample as technical replicates. For MDA-MB-231 tumors, the metastasis sites in which the cells showing bigger nuclei, forming clusters bigger than 3000 μm^2 were counted and quantified in the whole lung slide and the three technical replicates was averaged for each sample. For E0771 tumors, all metastatic nodes that are visible were counted and quantified in the whole lung slide.

Supplementary Figures and Legends

Table. S1. Known LCFA CoA binding proteins identified in the proteomic analysis

gene name	Forward Score	Reverse Score	Forward H /L	Reverse H/ L	Description
ACAD9	76.8	81.1	100	0.01	Acyl-CoA dehydrogenase family member 9, mitochondrial
ACADVL	66.9	75.5	9.7	0.01	Very long-chain acyl-CoA dehydrogenase, mitochondrial
ACBD1	36.3	35.5	16.5	0.01	Acyl-CoA-binding protein
ACBD5	64.0	67.3	12.1	0.01	Acyl-CoA-binding domain-containing protein 5

ACBD6	29.7	37.0	100	0.01	Acyl-CoA-binding domain-containing protein 6
ACBD7	13.4	20.2	100	0.01	Acyl-CoA-binding domain-containing protein 7
ACOT13	174.1	126.0	15.1	0.054	Acyl-coenzyme A thioesterase 13
ACOT7	120.8	79.6	8.6	0.067	Acyl-CoA thioesterase 7
ACOT9	4.3	5.6	100	0.01	Acyl-coenzyme A thioesterase 9, mitochondrial
ACOX1	55.4	46.4	100	0.01	Peroxisomal acyl-coenzyme A oxidase 1
FAR1	35.3	37.4	100	0.01	Fatty acyl-CoA reductase 1
HADHA	177.4	112.8	7.8	0.20	Trifunctional enzyme subunit alpha, mitochondrial
NMT1	21.1	33.2	100	0.01	Myristoyl-CoA:protein N-myristoyltransferase 1

Table. S2. Proteins identified in the proteomic analysis that were known to bind or be regulated by LCFA-CoA *in vitro*.

gene name	Forward Score	Revers e Score	Forward H /L	Revers e H/ L	Description	Ref
**ANT1**	50.8	49.8	3.7	0.01	ADP/ATP translocase 1	11-15
**ANT2**	59.0	62.8	3.8	0.21	ADP/ATP translocase 2	9-13
**ANT3**	55.1	58.4	100.0	0.22	ADP/ATP translocase 3	9-13
**FASN**	31.9	33.6	100.0	0.01	Fatty acid synthase	16
**GLUD1**	271.0	185.1	13.0	0.01	Glutamate dehydrogenase 1, mitochondrial	17
**IDH2**	154.7	99.7	15.6	0.01	Isocitrate dehydrogenase [NADP], mitochondrial	18
**PFKM**	141.7	107.5	1.5	0.01	ATP-dependent 6-phosphofructokinase	19
**PGD**	29.5	44.2	100.0	0.01	RecName: Full=6-phosphogluconate dehydrogenase	18

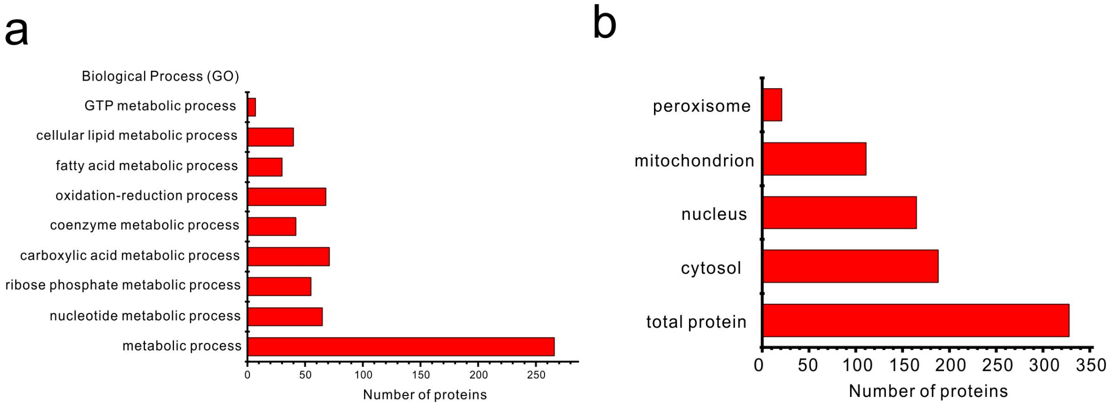

Fig. S1. Biological process analysis of LCFA-CoA interacting proteins (a) and subcellular distribution of LCFA CoA interacting proteins (b). Categories were assigned based on Biological Process (GO) analysis.

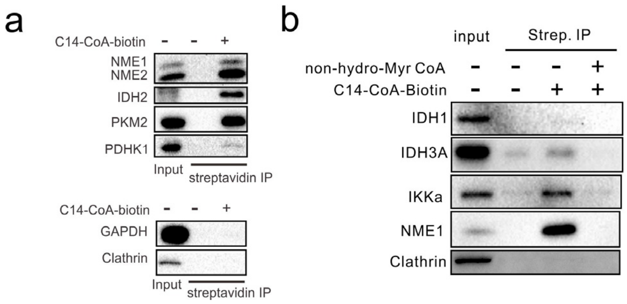

Fig. S2. Validation of some identified proteins by LCFA-CoA-biotin probe pull-down and western blot. (a) Pull down of the indicated proteins using C14-CoA-biotin in cell lysate. (b) Non-hydrolysable myristoyl CoA was able to compete with C14-CoA-biotin in the pull down.

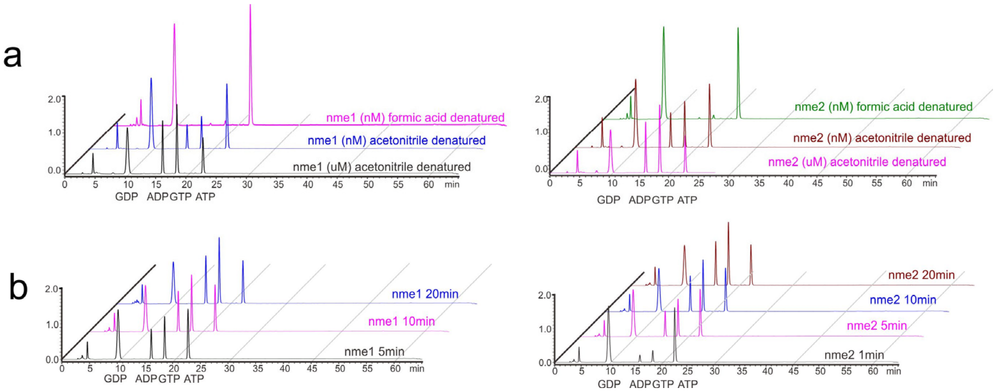

Fig. S3. Using an optimized HPLC assay to monitor the nucleoside diphosphate kinase (NDPK) activity directly. (a) Separation of different nucleotides and nucleosides. The NME1/2 proteins were effectively denatured with formic acid, while denaturation with acetonitrile was less effcient. (b) Monitoring the reactions at different time points.

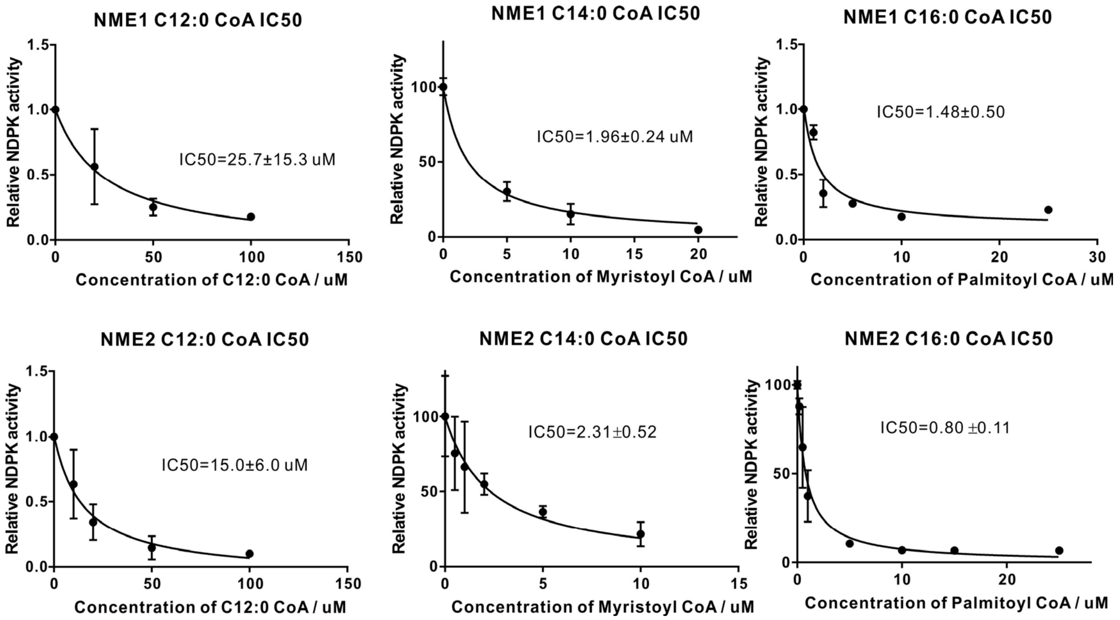

Fig. S4. IC50 value determination of C12:0, C14:0, and C16:0 LCFA-CoA on NME1 and NME2. For all graphs, the dots reflect the mean and the error bars represent standard deviation of 3 independent replicates.

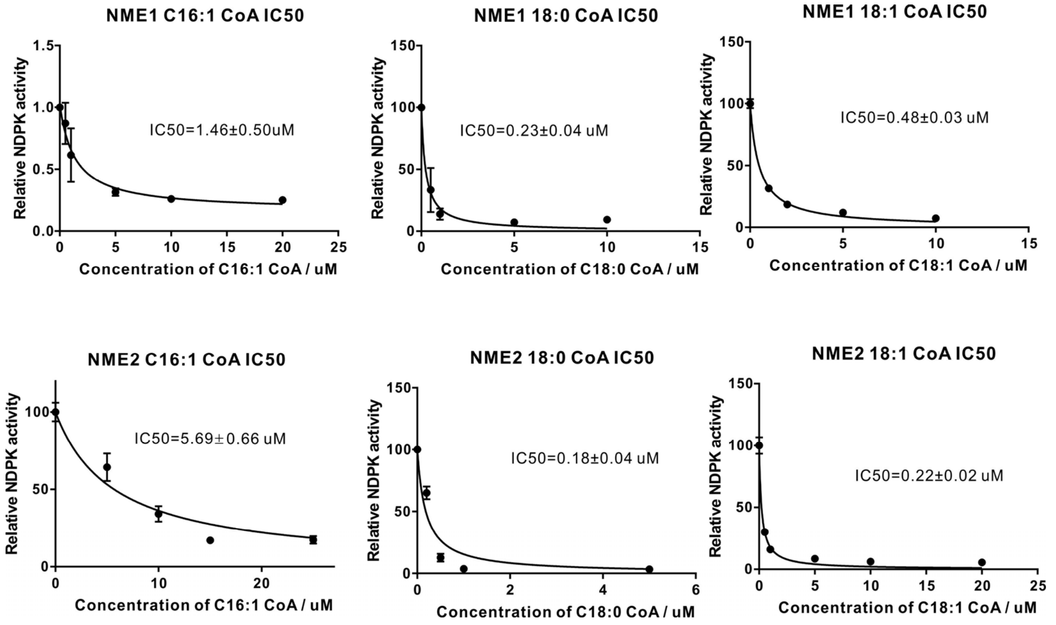

Fig. S5. IC50 value determination of C16:1, C18:0, and C18:1 LCFA-CoA on NME1 and NME2. For all graphs, the dots reflect the mean and the error bars represent standard deviation of 3 independent replicates.

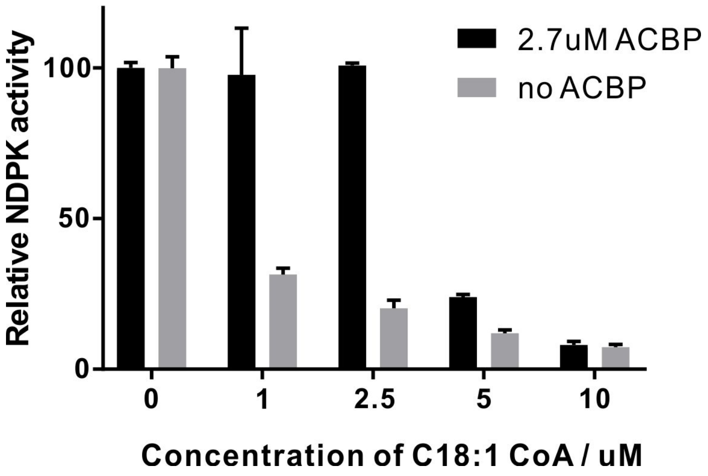

Fig. S6. The inhibition of NME1 by C18:1 CoA in the presence or absence of ACBP.

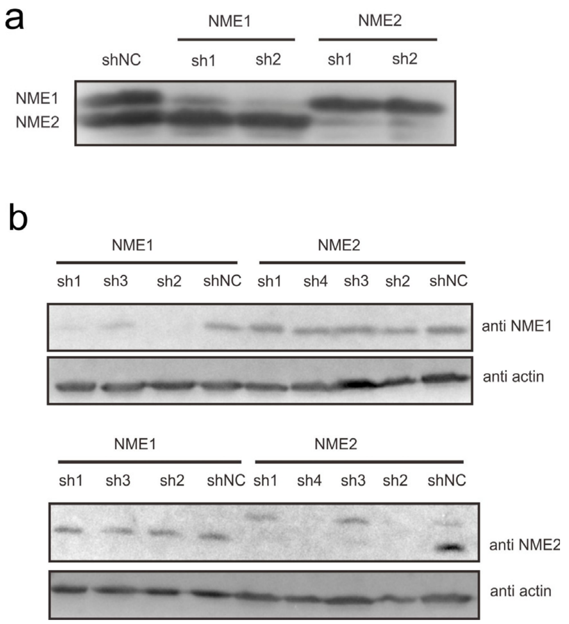

Fig. S7. Western blot images showing the knockdown of NME1/2 in Hela cells (a) and MDA-MB-231 cell (b).

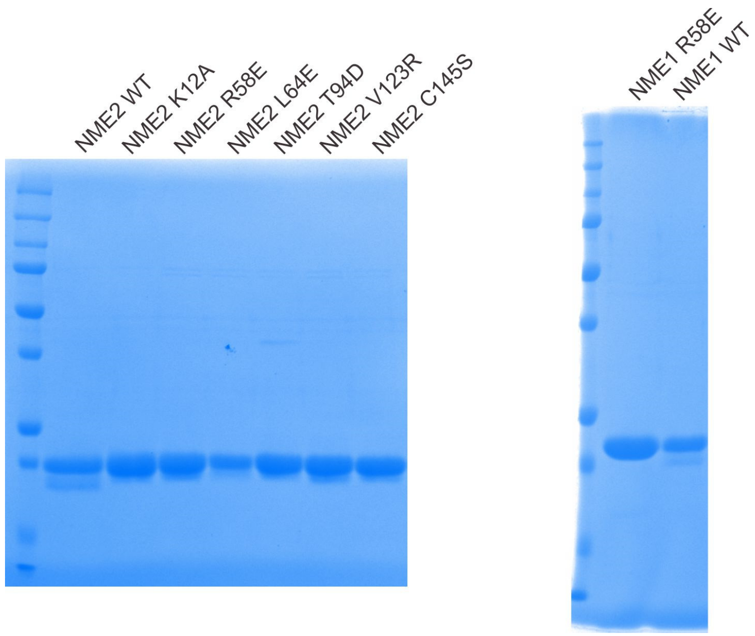

Fig. S8. Gel images showing the purity of NME1/2 WT and mutant proteins purified from *E coli*.

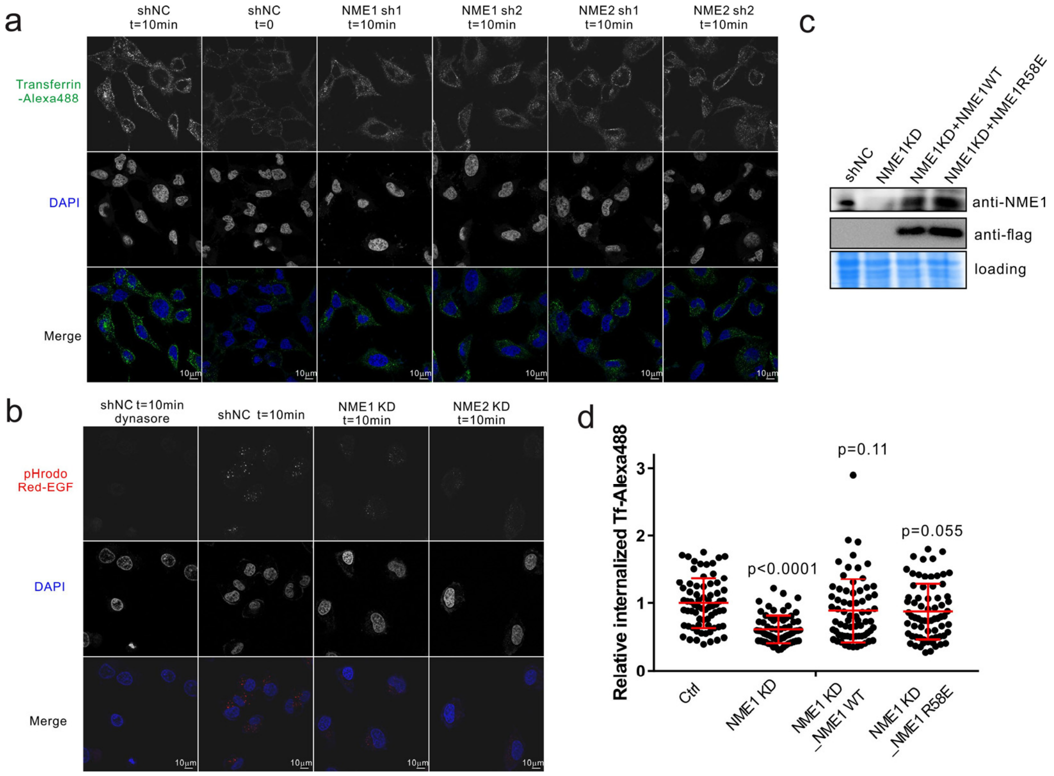

Fig. S9. The effect of NME1/2 knockdown on transferrin internalization (a) and EGF internalization (b) in Hela cells without oleic acid treatment monitored by confocal microscopy. Scale bars, 10 μm. The endocytic defect of transferrin receptor was rescued

in Hela cells by expression of flag-tagged wild-type or R58E mutant, shRNA-resistant NME1 (c, d). NME1 was stably knocked with shRNA#3, which targets the 3'UTR. Transferrin internalization was monitored and quantified by Cytation 5 imaging station. Statistical significance was calculated by unpaired two-tailed Student's t test.

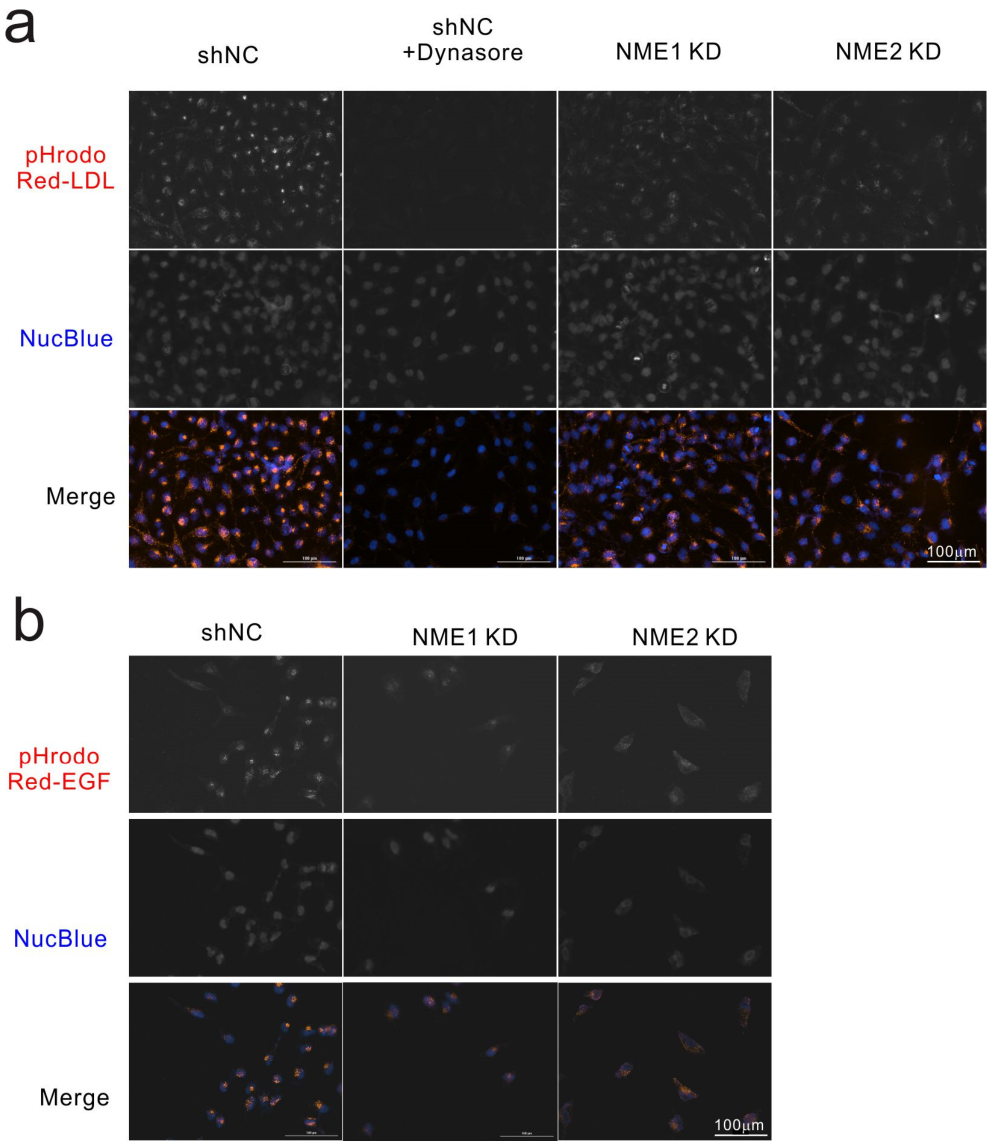

Fig. S10. The effect of NME1/2 knockdown on (a) LDL internalization and (b) EGF internalization in Hela cells monitored by Cytation 5 imaging station. Scale bars, 100 μm

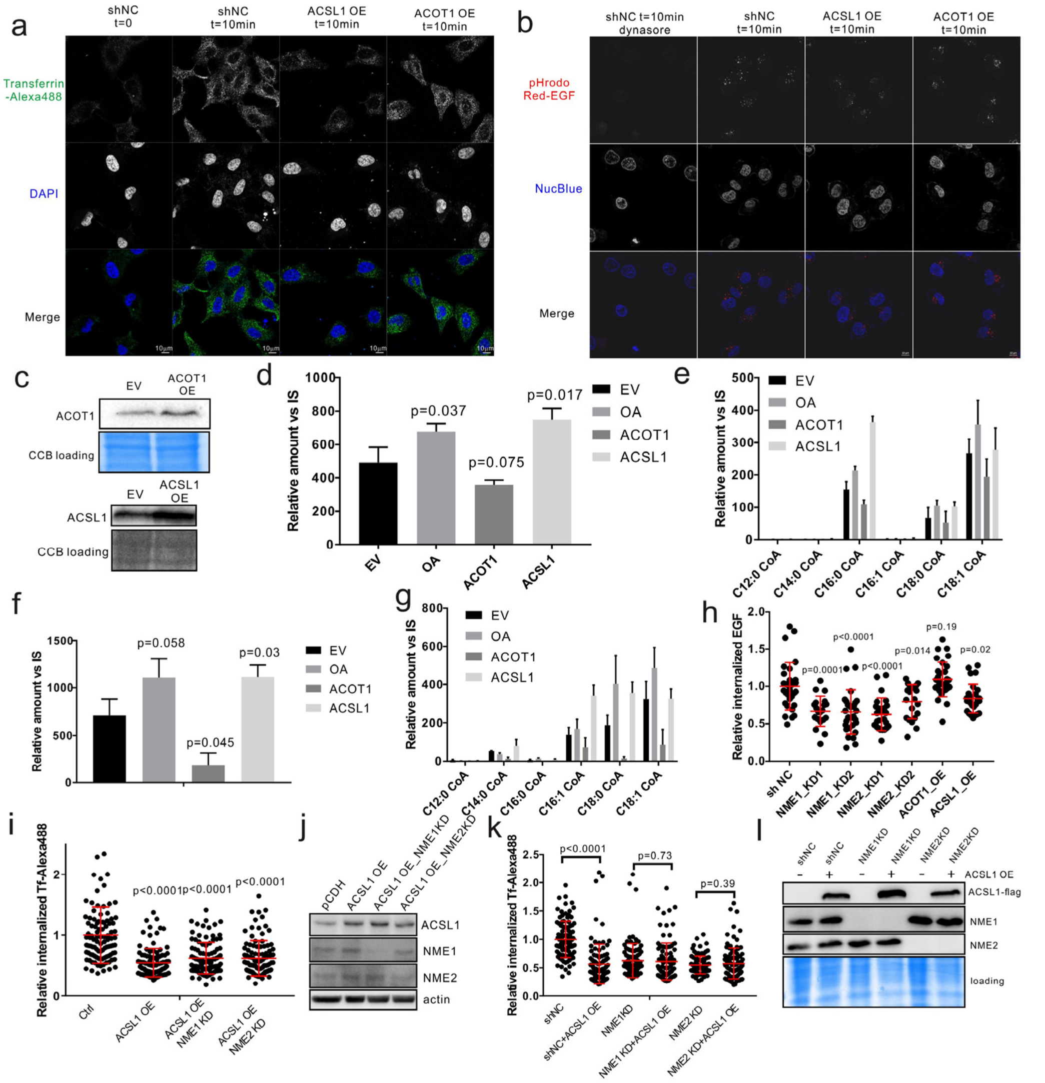

Fig. S11. The effect of ACSL1 or ACOT1 overexpression on transferrin internalization (a) and EGF internalization (b) in Hela cells monitored by confocal microscopy. The overexpression of ACSL1 and ACOT1 were checked by western blot (c). The relative amounts of total LCFA CoAs (C14:0 CoA, C16:0 CoA, C16:1 CoA, C18:0 CoA and C18:1 CoA) that measured in our experiments were quantified by targeted metabolomic analysis in Hela cells (d) and MDA-MB-231 cells (f). The changes of different LCFA CoAs in Hela cells (e) and MDA-MB-231 cells (g) were shown as well. The amount shown is relative to the internal standard, C17:0 CoA. The bar heights reflect the mean and the error bars represent standard deviation of 3 independent biological replicates. Quantification of EGF internalization on Cytaion 5 is shown (n=30 cells) (h). The effect of NME1/2 knockdown on Tf internalization in the background of ACSL1 overexpression (i, j). The effect of ACSL1 overexpression on Tf internalization in the

background of NME1/2 knockdown (k, l). Statistical significance was calculated by unpaired two-tailed Student's t test. Scale bars, 10 μm.

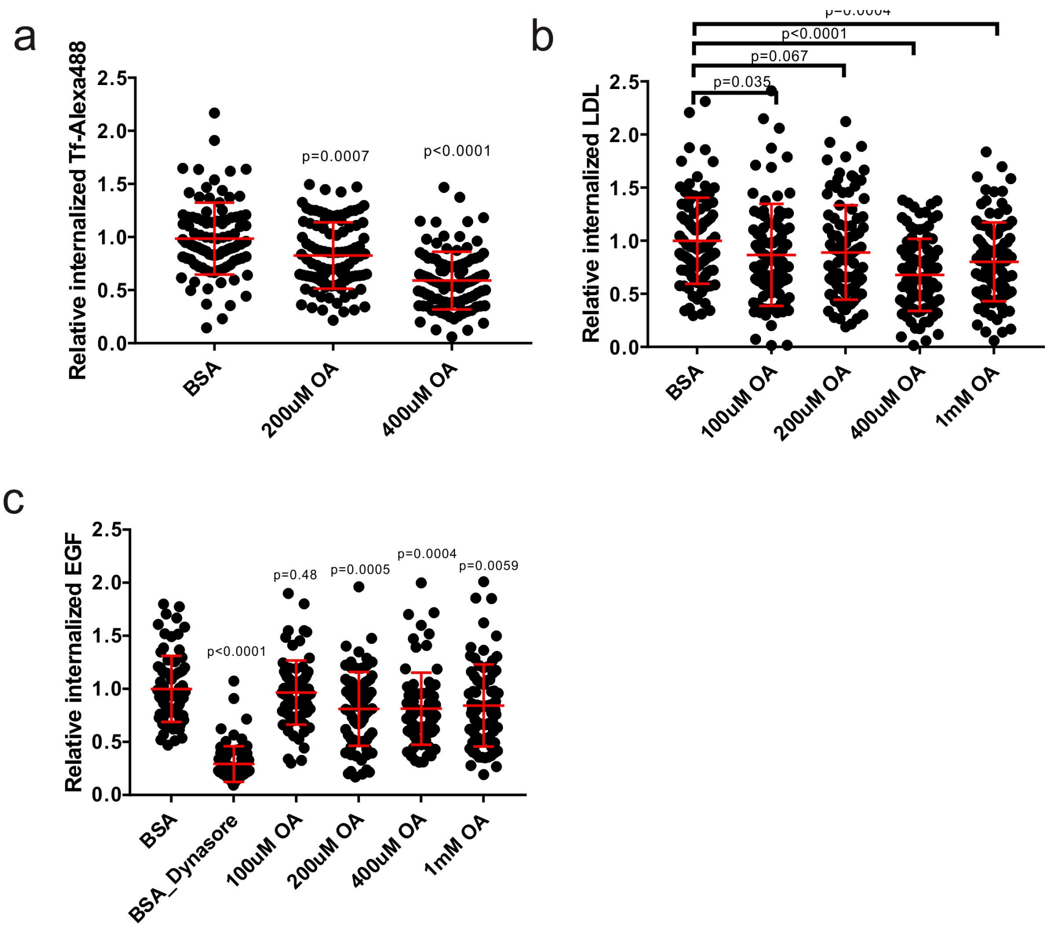

Fig. S12. Quantification of the effects of 3-h oleic acid treatment on transferrin internalization (n=100 cells) (a), LDL internalization (n=100 cells) (b), and EGF internalization (n=80 cells) (c) in Hela cells monitored by Cytaion 5. Statistical significance was calculated by unpaired two-tailed Student's t test. Data shown in the figure are representative of minimally two independent experiments.

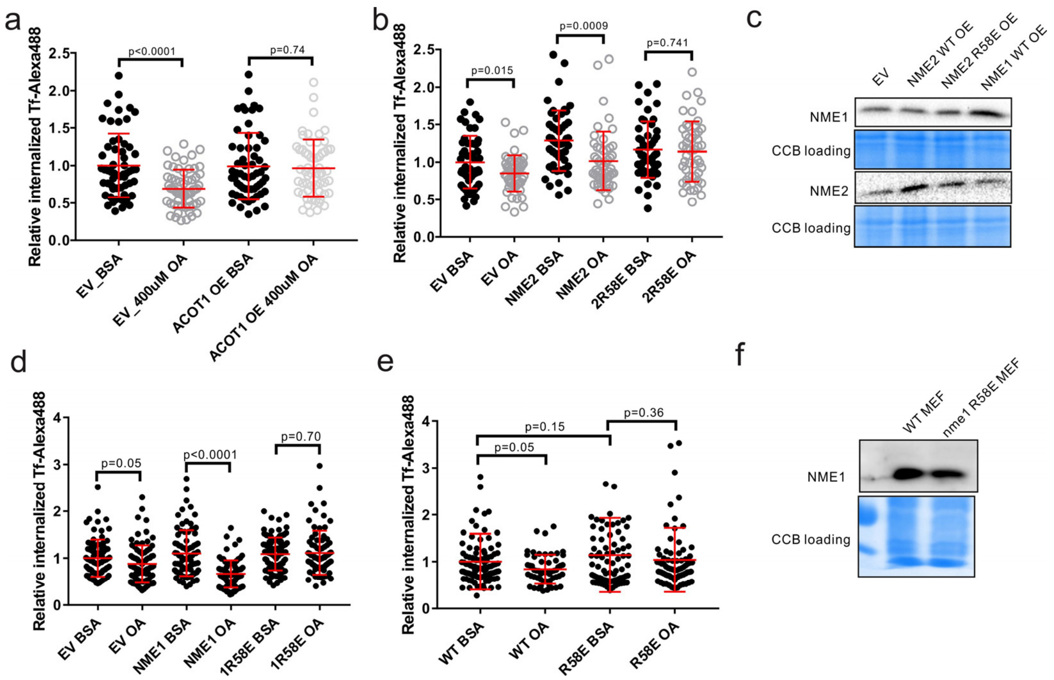

Fig. S13. Quantification of the effect of 3-h oleic acid treatment (400 µM) on transferrin internalization with or without ACOT1 overexpression (n = 60 cells) (a) and with or without NME2 WT/mutant overexpression (n=50 cells) (b) in Hela cells monitored by Cytaion 5. (c) The overexpression of NME2 WT, NME2 R58E, and NME1 WT in Hela cells was checked by western blot. Statistical significance was calculated by unpaired two-tailed Student's t test. Quantification of the effect of 3-h oleic acid treatment (250 µM) on transferrin internalization in (d) MDA-MB-231 cells stably expressing empty vector, NME1 WT or NME1 R58E and (e) NME1 WT or R58E MEF cells. (f) NME1 protein level in MEF cells. Data shown in the figure are representative of minimally two independent experiments.

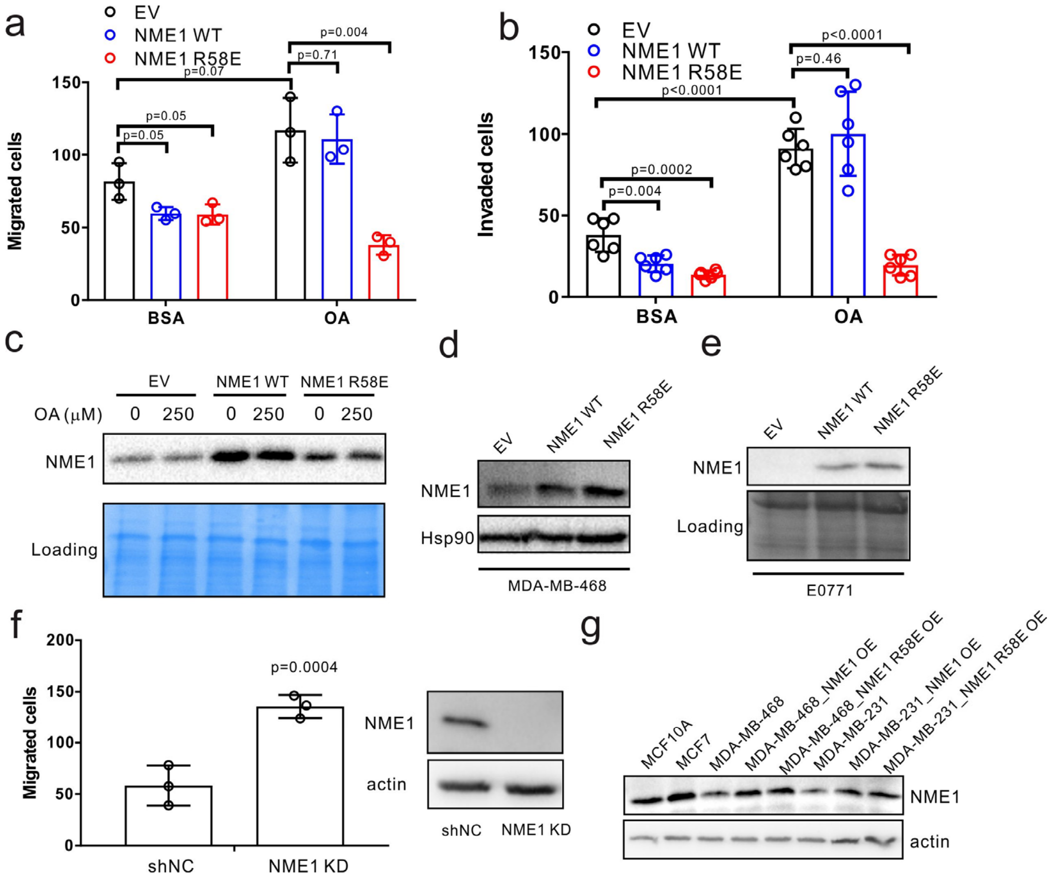

Fig. S14. The effect of NME1 on breast cancer migration and invasion. (a) Boyden chamber migration assay for MDA-MB-468 cells. (b) Invasion assay for MDA-MB-231 cells. Stably overexpression of NME1 WT or R58E in (c) MDA-MB-231, (d) MDA-MB-468 and (e) E0771 cells. (f) Boyden chamber migration assay for MDA-MB-468 cells with NME1 stably knockdown. (g) NME1 level in non-malignant breast epithelial cells (MCF10A), non-metastatic breast cancer cell (MCF7) and metastatic cancer cell lines used in this study. NME1 was detected with NME1 antibody. Statistical significance was calculated by unpaired two-tailed Student's t test. For all graphs, the middle lines reflect the mean and the error bars represent standard deviation of independent biological replicates.

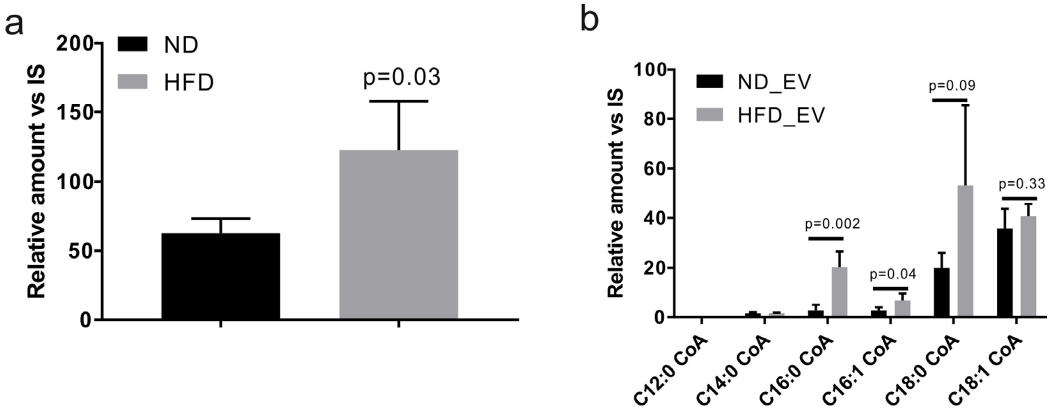

Fig. S15. The effect of diet on intracellular major LCFA-CoA species levels in breast tumors in mouse model. Total amount of the LCFA-CoAs (C14:0, C16:0, C16:1, C18:0 and C18:1) tested in (a) MDA-MB-231 tumors (n=4) in NSG mice. The amount shown is relative to the internal standard, C17:0 CoA. The changes of each LCFA-CoA are shown in (b). Statistical significance was calculated by unpaired two-tailed Student's t test. For all graphs, the middle lines/bar heights reflect the mean and the error bars represent standard deviation of independent biological replicates.

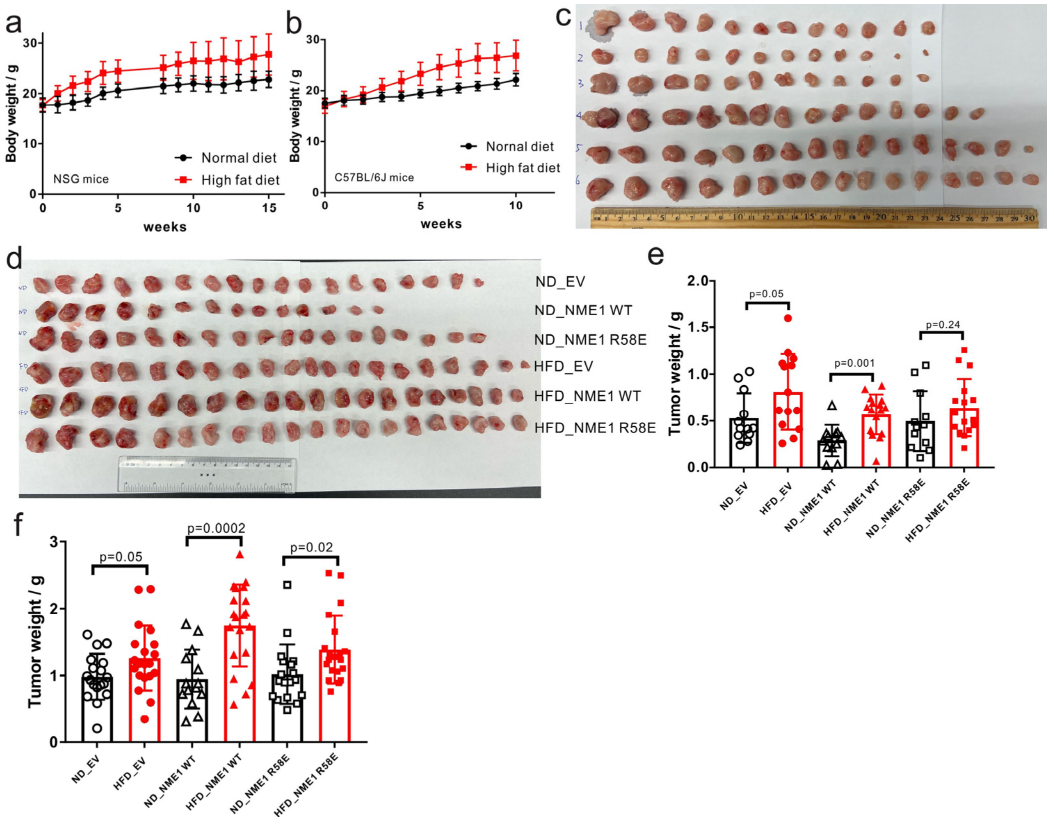

Fig. S16. The effect of HFD on primary tumor growth. The body weights of the mice

fed with either normal diet or high-fat diet for (a) NSG mice and (b) C57BL/6J mice. The primary tumors obtained from (c) NSG mice and (d) C57BL/6J mice fed with normal diet or high-fat diet. The quantification of tumor weights from (e) NSG mice and (f) C57BL/6J mice. No statistical significance between ND_EV vs ND_NME1 WT or ND_EV vs ND_NME1 R58E in both (e) and (f). Statistical significance was calculated by unpaired two-tailed Student's t test. For all graphs, the middle lines/bar heights reflect the mean and the error bars represent standard deviation of independent biological replicates.

References:

1. Lehmann, J., Richers, J., Pothig, A. & Sieber, S.A. Synthesis of ramariolide natural products and discovery of their targets in mycobacteria. *Chem Commun* **53**, 107-110 (2017).
2. Wang, L. *et al.* Synthesis and Antitumor Activity of a Novel Series of 6-Substituted Pyrrolo[2,3-d]pyrimidine Thienoyl Antifolate Inhibitors of Purine Biosynthesis with Selectivity for High Affinity Folate Receptors and the Proton-Coupled Folate Transporter over the Reduced Folate Carrier for Cellular Entry. *J Med Chem* **53**, 1306-1318 (2010).
3. Zhang, X.Y., Cao, J., Miller, S.P., Jing, H. & Lin, H.N. Comparative Nucleotide-Dependent Interactome Analysis Reveals Shared and Differential Properties of KRas4a and KRas4b. *Acs Central Sci* **4**, 71-80 (2018).
4. Chapter 11.1 Nucleosides and nucleotides, in *Laboratory Techniques in Biochemistry and Molecular Biology*, Vol. 17. (eds. A. Fallon, R.F.G. Booth & L.D. Bell) 144-168 (Elsevier, 1987).
5. Kabsch, W. Xds. *Acta Crystallogr D* **66**, 125-132 (2010).
6. Evans, P.R. & Murshudov, G.N. How good are my data and what is the resolution? *Acta Crystallogr D* **69**, 1204-1214 (2013).
7. Mccoy, A.J. *et al.* Phaser crystallographic software. *J Appl Crystallogr* **40**, 658-674 (2007).

8. Dexheimer, T.S. *et al.* NM23-H2 may play an indirect role in transcriptional activation of c-myc gene expression but does not cleave the nuclease hypersensitive element III1. *Mol Cancer Ther* **8**, 1363-1377 (2009).
9. Emsley, P., Lohkamp, B., Scott, W.G. & Cowtan, K. Features and development of Coot. *Acta Crystallogr D* **66**, 486-501 (2010).
10. Liebschner, D. *et al.* Macromolecular structure determination using X-rays, neutrons and electrons: recent developments in Phenix. *Acta Crystallographica Section D-Structural Biology* **75**, 861-877 (2019).
11. Shug, A., Lerner, E., Elson, C. & Shrago, E. The inhibition of adenine nucleotide translocase activity by oleoyl CoA and its reversal in rat liver mitochondria. *Biochem Biophys Res Commun* **43**, 557-563 (1971).
12. Harris, R.A., Farmer, B. & Ozawa, T. Inhibition of the mitochondrial adenine nucleotide transport system by oleyl CoA. *Arch Biochem Biophys* **150**, 199-209 (1972).
13. Ciapaite, J. *et al.* Metabolic control of mitochondrial properties by adenine nucleotide translocator determines palmitoyl-CoA effects. Implications for a mechanism linking obesity and type 2 diabetes. *FEBS J* **273**, 5288-5302 (2006).
14. Ciapaite, J. *et al.* Modular kinetic analysis of the adenine nucleotide translocator-mediated effects of palmitoyl-CoA on the oxidative phosphorylation in isolated rat liver mitochondria. *Diabetes* **54**, 944-951 (2005).
15. Woldegiorgis, G., Shrago, E., Gipp, J. & Yatvin, M. Fatty acyl coenzyme A-sensitive adenine nucleotide transport in a reconstituted liposome system. *J Biol Chem* **256**, 12297-12300 (1981).
16. Lust, G. & Lynen, F. The inhibition of the fatty acid synthetase multienzyme complex of yeast by long-chain acyl coenzyme A compounds. *Eur J Biochem* **7**, 68-72 (1968).
17. Kawaguchi, A. & Bloch, K. Inhibition of glutamate dehydrogenase and malate dehydrogenases by palmitoyl coenzyme A. *J Biol Chem* **251**, 1406-1412 (1976).
18. Taketa, K. & Pogell, B.M. The effect of palmityl coenzyme A on glucose 6-phosphate dehydrogenase and other enzymes. *J Biol Chem* **241**, 720-726

(1966).

19. Jenkins, C.M., Yang, J., Sims, H.F. & Gross, R.W. Reversible high affinity inhibition of phosphofructokinase-1 by acyl-CoA: a mechanism integrating glycolytic flux with lipid metabolism. *J Biol Chem* **286**, 11937-11950 (2011).
